# Fungal-derived methyldeoxaphomins target *Plasmodium falciparum* segregation through the inhibition of PfActin1

**DOI:** 10.1073/pnas.2418871122

**Published:** 2025-02-18

**Authors:** Tiantian Jiang, Jin Woo Lee, Jennifer E. Collins, Samuel Schaefer, Daisy Chen, Flore Nardella, Karen Wendt, Thilini G. Peramuna, Raphaella Paes, James L. McLellan, Jasveen Bhasin, Gregory L. Durst, Kirsten K. Hanson, Debopam Chakrabarti, Robert H. Cichewicz, Elizabeth A. Winzeler

**Affiliations:** ^a^Department of Pediatrics, School of Medicine, University of California, San Diego, CA 92093; ^b^College of Pharmacy, Duksung Women’s University, Seoul 01369, Republic of Korea; ^c^Division of Molecular Microbiology, Burnett School of Biomedical Sciences, University of Central Florida, Orlando, FL 32826; ^d^Department of Medicinal Chemistry, College of Pharmacy, University of Michigan, Ann Arbor, MI 48109; ^e^Department of Molecular Microbiology and Immunology, South Texas Center for Emerging Infectious Diseases, University of Texas at San Antonio, San Antonio, TX 78249; ^f^Lgenia Inc., Fortville, IN 46040; ^g^Skaggs School of Pharmacy and Pharmaceutical Sciences, University of California, San Diego, CA 92093

**Keywords:** *Plasmodium falciparum*, fungi-derived natural product, methyldeoxaphomin

## Abstract

Herein we report the finding and structure determination of a natural product based on the methyldeoxaphomin scaffold family from the fungus *Trichocladium asperum* that shows promising antiplasmodial activity and selectivity against host cells. In vitro evolution and whole genome analysis in *Plasmodium falciparum* with the most potent member, NPDG-F (EC_50_ of 550 nM in Dd2; 290 nM in 3D7), shows that parasite resistance to methyldeoxaphomins is strongly associated with mutations in PfActin1 (PF3D7_1246200), a critically essential ATPase needed for all stages of parasite development. Molecular docking study with available PfActin1 crystal structure shows NPDG-F occupies the same allosteric binding pocket as the known actin inhibitor cytochalasin D. The direct PfActin1 target engagement in the allosteric site was supported by cross-resistance studies, isobologram analysis with other PfActin1 inhibitors, and the structure–activity relationships for the methyldeoxaphomin family. When added to in vitro culture, NPDG-F induced morphological abnormalities in merozoite cellularization during schizogony in both the *Plasmodium* blood and liver stages. Our data provide chemical validation that PfActin1 is an attractive, pan-lifecycle target and inform strategies for the design of more selective inhibitors.

## Significance

Malaria remains a deadly disease that claims the lives of hundreds of thousands each year. Extracts from living organisms have been a traditional source of antimalarial remedies and the basis for the two most widely used modern medicines, aminoquinolines and endoperoxides. Here, we describe our finding and characterization of the active antimalarial ingredient in an extract derived from a common soil fungus. By determining how this compound works in the malaria parasites, we identified a molecular vulnerability that may assist in the design of future drugs.

Natural products have consistently been a rich source of potent, life-saving antimicrobials (1). The impact of natural products or natural product-derived compounds in the field of malaria therapeutics has been—and arguably remains—an incredible success story, with drugs like artemisinin, quinidine, chloroquine, artemether, artesunate, and many others stemming from natural products. Together, these medicines have helped to combat the disease and profoundly reduce early childhood mortality in many parts of the world where malaria remains endemic (2). Despite this success, natural products face limitations such as often being difficult to isolate, expensive to produce, or lacking drug-like properties. However, investigations into the mechanism of action of these natural products can aid the exploration of new chemically validated, druggable targets that can be used in the discovery and development of modern medicines that are less toxic, inexpensive to produce, and more orally bioavailable.

Of the natural product sources available, fungi contain a rich wellspring of bioactive compounds that can be explored for their antimicrobial potential or used as molecular probes for target discovery. Many important compound classes of natural products have been identified from fungi and investigated for their medicinal benefits (3). For example, fungal polypeptides such as efrapeptins, zervamicins, and antiamoebin were found to exhibit potent antiparasitic and anticancer activities (4). More recently, a large-scale, comprehensive natural product discovery effort involving a screen of 7,149 fungi extracts was conducted, revealing numerous scaffolds with antiplasmodial potential (5). One such scaffold is xanthoquinodin from *Trichocladium sp.* which has shown potent inhibition against *Plasmodium falciparum* (6), *Trichomonas vaginalis*, *Cryptosporidium parvum*, *Mycoplasma genitalium* (7), and *Toxoplasma gondii* (8). Other fungal natural products highlighted for possessing potent, dual activity against these two closely related apicomplexans (*T. gondii* and *P. falciparum*) were peptaibols, cyclic tetrapeptides, heptalidic acid analogs, and fumagillin analogs (8–10).

As part of an on-going multi-institute collaboration to identify new antiplasmodial compounds from the University of Oklahoma fungal extract collection, we communicated the identification of 5 xanthoquinodin compounds (three known, two unknown) with antiplasmodial activity isolated from the fungus *Trichocladium asperum* (7). In addition to these 5 inhibitors, 6 metabolites structurally similar to cytochalasin D were also isolated from this fungus. Here, we describe their antiplasmodial activity, structure determination, and mode of action. Unlike the cytochalasins, these 6 analogs (later named methyldeoxaphomins NPDG-A to F) do not contain an ether linkage between carbons 23 and 9, making them more synonymous with deoxaphomins (11). These compounds constitute a different class of actin polymerization inhibitors.

## Results

### Structure Elucidation of Methyldeoxaphomin Analogs from *Trichocladium asperum*.

**Fig. 1.**
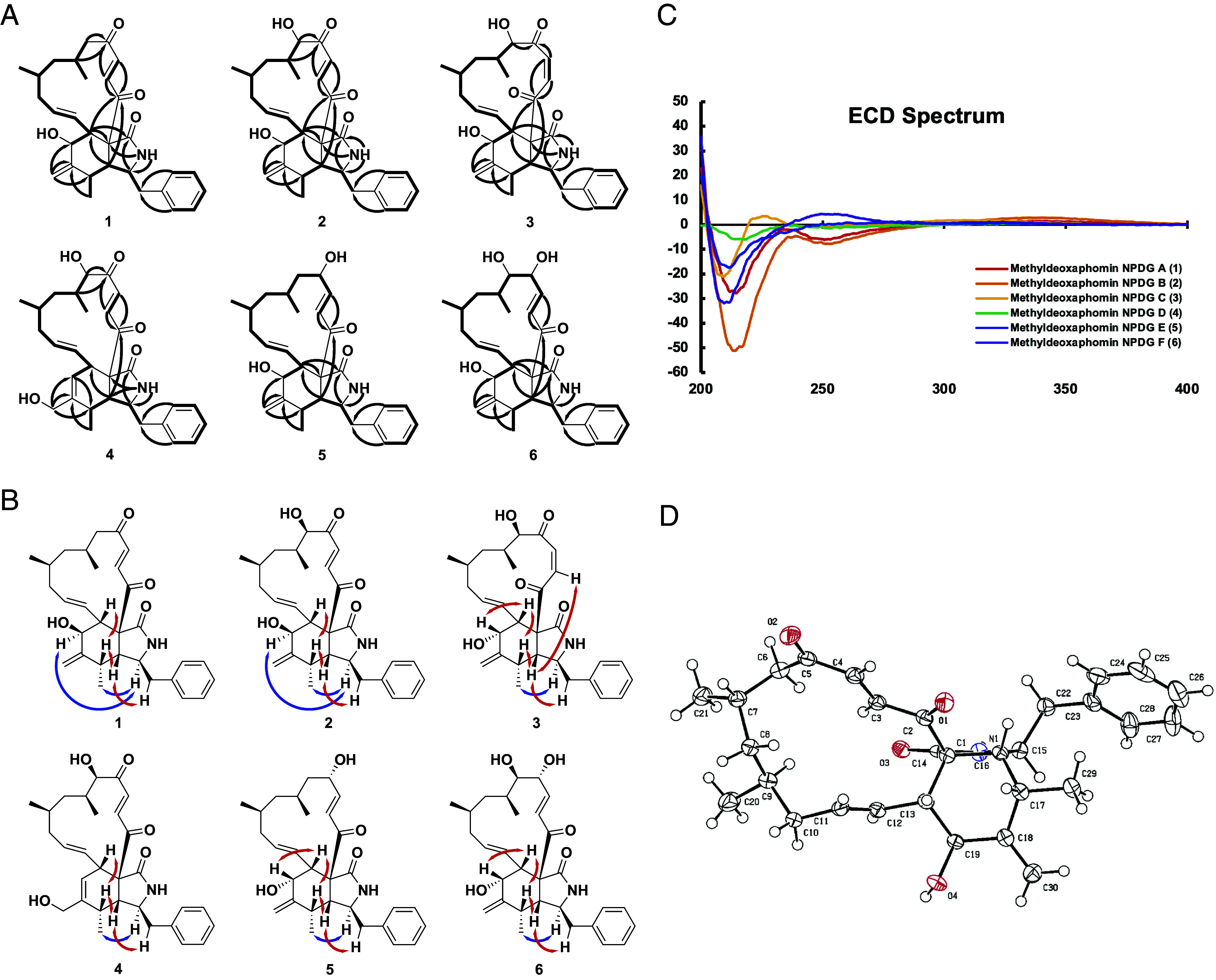
Structural dissection of the methyldeoxaphomin analogs from *T. asperum.* (*A*) Key correlations of HMBC (→) and ^1^H–^1^H COSY (—) of compounds 1 to 6. (*B*) Key correlations of ^1^H–^1^H ROESY (↔) in the isoindole core of compounds 1-6 (red double arrows: b-orientation; blue double arrows: a-orientation). (*C*) ECD spectrum of methyldeoxaphomin NPDG-A to F (compound 1 to 6). (*D*) X-ray ORTEP plot for the molecular structure of compound 1. The 3D structures of the compound 2 to 6 were deduced from the crystal structure of compound 1.

The *Trichocladium sp.* fungus processed in this research (TN09213 RBM-1) was originally isolated from a soil sample collected by a citizen scientist near Lebanon, Tennessee, in the USA. Its activity-guided dereplication and fractionation was prompted by the previous identification of antiplasmodial activity in crude extracts of *Trichocladium asperum.* Following these efforts, we identified six active metabolites (1–6). Their structures were determined based on the interpretation of 1D (^1^H and ^13^C) and 2D NMR spectroscopic data (HSQC: heteronuclear single quantum coherence; HMBC: heteronuclear multiple bond correlation; COSY: correlation spectroscopy, and ROESY: rotating-frame Overhauser Effect spectroscopy) (*SI Appendix*, Tables S1 and S2) and data from HRESIMS (high-resolution electrospray ionization mass spectrometry) (Fig. 1 *A* and *B*). Further, their absolute configurations were elucidated by ECD (electronic circular dichroism) experiment and chemical reactions such as Mosher’s method (Fig. 1*C*). Single-crystal X-ray diffraction analysis further confirmed the absolute configurations of these metabolites (Fig. 1*D*).

These compounds exhibit structural similarities to both cytochalasin and deoxaphomin analogs, yet they align more closely with deoxaphomins due to their 13-membered carbon ring, contrasting the 14-membered macrocyclic ring present in cytochalasins (cytochalasin D being an exception). Distinguishing themselves from deoxaphomins, these compounds feature a methyl group at the sixth carbon position of the ring, leading to their designation as methyldeoxaphomin.

### Methyldeoxaphomin Analogs Exhibit Antiplasmodial Activities.

The antiplasmodial activity of these analogs was assessed in *P. falciparum* asexual blood stage using a SYBR Green I-based assay. Compounds were tested in both the multidrug resistant line Dd2, and the chloroquine-sensitive line 3D7, to probe any potential for preexisting resistance. No substantial difference in activity between the two cell lines was observed, suggesting a distinct target from chloroquine and related analogs (Table 1 and Dataset S1). These methyldeoxaphomin analogs were also cross-screened for cytotoxicity in the human cell line HepG2 to determine their selectivity for the *Plasmodium* parasite. Despite the small number of analogs, a clear shift in activity was noted with R_1_ group changes. Substitution from H to OH at carbon 19 resulted in antiplasmodial activity in a previously inactive compound, as seen with methyldeoxaphomins NPDG-B (Dd2 EC_50_ = 1.5 µM) and NPDG- A (Dd2 EC_50_ > 5 µM). Cytotoxicity increased in HepG2 as well, with no inhibition seen at the highest concentrations tested with NPDG-A (25 µM), and a HepG2 EC_50_ of 9.5 µM with NPDG-B. Similarly, a R_2_ group change from O to OH at carbon 20 resulted in a lower magnitude of increase in antiplasmodial activity, as seen with methyldeoxaphomin NPDG-E (Dd2 EC_50_ = 2.6 µM). Intriguingly, unlike NPDG-B, very minimal cytotoxicity was noted with this substitution, with NPDG-E having a HepG2 EC_50_ of only 23 µM. This effect was maintained with analog NPDG-F where both R_1_ and R_2_ were substituted with OH groups. The analog NPDG-F demonstrated the greatest antiplasmodial potency with a Dd2 EC_50_ of 0.55 µM and no signs of cytotoxicity at 25 µM. The remaining analogs NPDG-C and NPDG-D showed Dd2 EC_50_s of 0.68 and > 5 µM, respectively. Both inhibitors showed cytotoxicity in line with NPDG-B, with EC_50_s of 8.1 and 8.2 µM in HepG2 for NPDG-C and NPDG-D, respectively. It is worth noting that both analogs maintained an O group at carbon 20, suggesting that the OH substitution seen in this location may be key to increased selectivity. In addition, the hydroxyl group at carbon 7 seems to be critical for function as a drastic loss of potency was found in NPDG-D as compared to B and C.

**Table 1.** Antiplasmodial activity of methyldeoxaphomin analogs

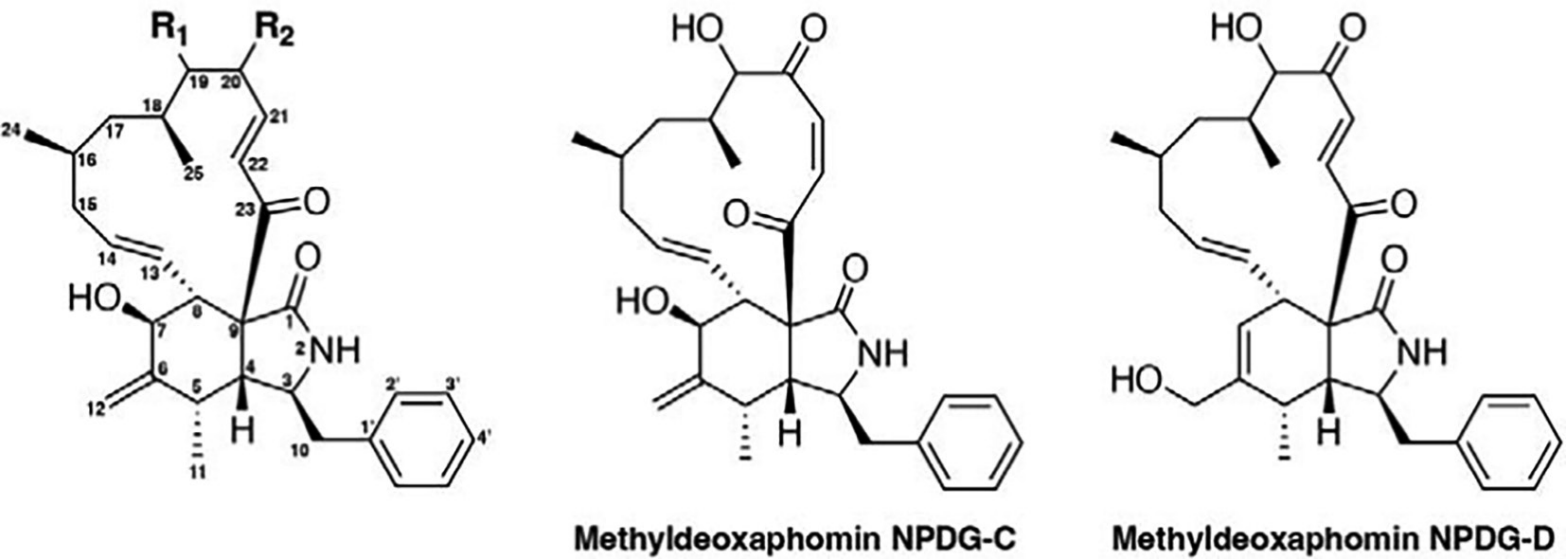						
ID	R_1_	R_2_	Dd2 EC_50_ (µM)	3D7 EC_50_ (µM)	HepG2 EC_50_ (µM)	SI
Methyldeoxaphomin NPDG-A	H	O	>5.0	>5.0	>25	−
Methyldeoxaphomin NPDG-B	OH	O	1.5 ± 0.16	1.7 ± 0.09	9.4 ± 0.27	5
Methyldeoxaphomin NPDG-C	OH	O	0.68 ± 0.19	0.84 ± 0.04	8.1 ± 0.23	11
Methyldeoxaphomin NPDG-D	OH	O	>5	>5	8.2 ± 0.07	−
Methyldeoxaphomin NPDG-E	H	OH	2.6 ± 0.31	2.8 ± 0.14	23 ± 0.92	9
Methyldeoxaphomin NPDG-F	OH	OH	0.55 ± 0.19	0.29 ± 0.03	>25	60

Results are expressed as means from triplicate experiments ± SEM. Selectivity Index (SI) = HepG2 EC_50_/($\bar{x}$: Dd2 EC_50_, 3D7 EC_50_).

### Methyldeoxaphomin NPDG-F Displays a Gradual Killing Profile in *P. falciparum*.

To further characterize the most potent compound among the analogs, methyldeoxaphomin NPDG-F, we determined its parasite reduction ratio (PRR) in the *P. falciparum* (12). Parasites were synchronized and exposed to a 10 × EC_50_ concentration of NPDG-F, fast-acting control dihydroartemisinin (DHA), or slow-acting control atovaquone for 24, 48, 72, 96, or 120 h. As shown in *SI Appendix*, Fig. S1*A*, NPDG-F possesses a gradual killing profile, in line with the inhibitor atovaquone. After a 24 h lag phase, only 10^0.64^ parasites are killed within the first replication cycle (48 h). As with atovaquone, more than 96 h are required for a 99.9% clearance of parasites. We also examined the in vitro killing rate in Dd2 through flow cytometric analysis. Parasites were incubated with 10 × EC_50_ concentration of NPDG-F, DHA, atovaquone, or a vehicle control for 12, 24, or 48 h. These results further confirmed that NPDG-F has a delayed inhibition phenotype and a cytocidal killing profile (*SI Appendix*, Fig. S1 *B*–*D*).

### The In Vitro Evolution of Resistance to NPDG-F Identifies PfActin1 as a Putative Drug Target.

**Fig. 2.**
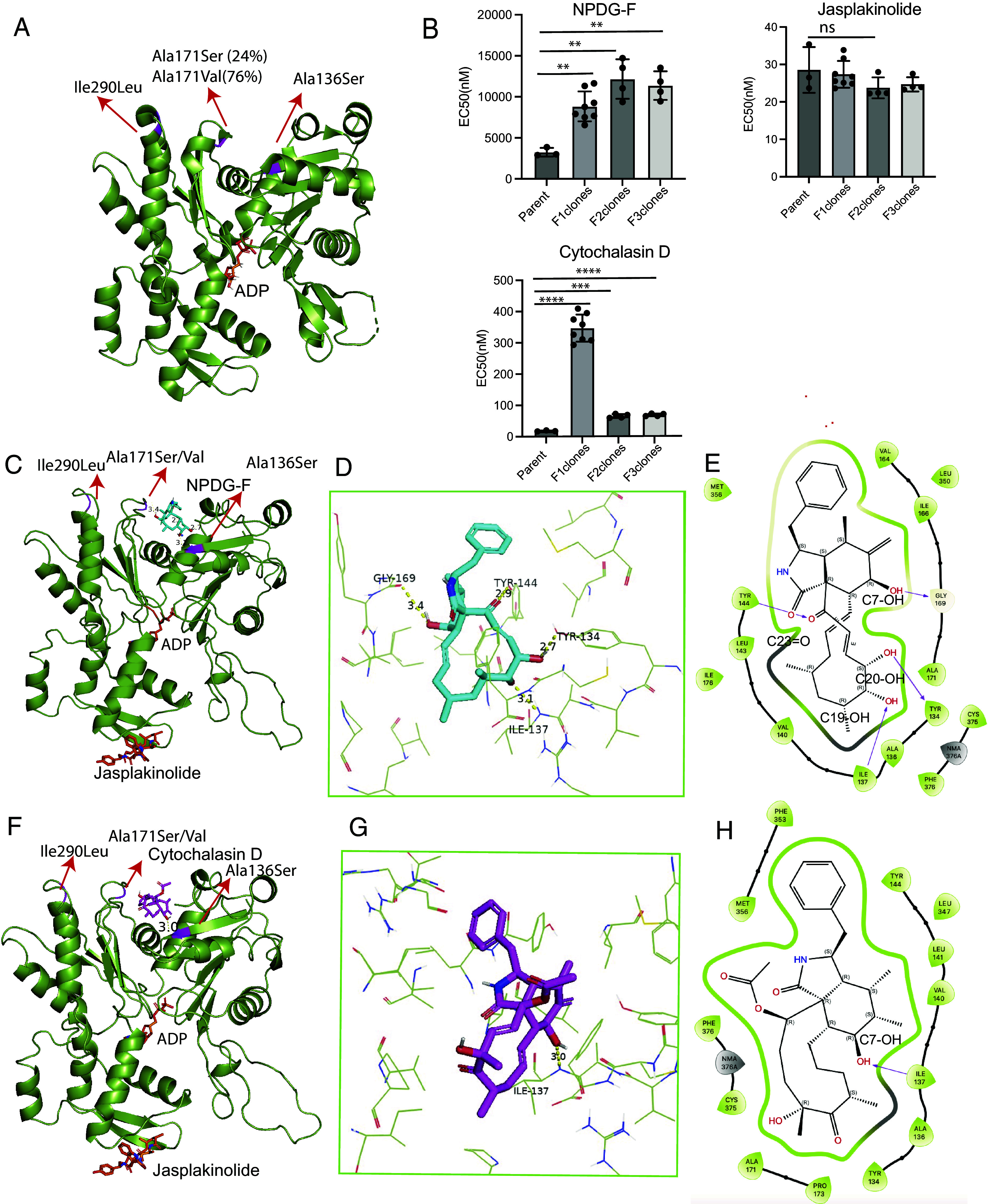
Identification of PfActin1 mutations through in vitro evolution and subsequent docking studies. (*A*) Mapping of missense mutations found through in vitro evolution and whole genome sequencing onto the 3D structure of PfActin1 (PDB ID: 6TU4). (*B*) Phenotyping of the resistant clones and their cross sensitivity to cytochalasin D and NPDG-F (**P ≤ 0.01; ***P ≤ 0.001; ns: not significant). (*C*) NPDG-F docked to PfActin1(PDB ID: 6TU4) containing ADP and jasplakinolide. The mutations (A136S, A171V/S, and I290L) are marked in magenta. (*D* and *E*) Close-up views of the binding pocket of NPDG-F in PfActin1, shown in 3D and 2D representations, respectively. (*F*) Cytochalasin D docked to PfActin1(PDB ID: 6TU4) containing ADP and jasplakinolide. The mutations (A136S, A171V/S, and I290L) are marked in magenta. (*G* and *H*) Close-up views of the binding pocket of cytochalasin D in PfActin1, shown in 3D and 2D representations, respectively.

We next sought to identify the molecular target of NPDG-F using in vitro evolution in a hypermutator line Dd2-Pol δ. This line was created to facilitate faster resistance development through the modification of the catalytic residues in DNA polymerase δ via CRISPR-Cas9 (13). Three populations of parasites grown in parallel were exposed to increasing concentrations of NPDG-F from the initial 90 nM to a final concentration of 17 μM over 6.3 mo. Once resistance (~4.2-fold) was identified in the 3 bulk culture populations, parasite DNA was extracted, followed by library preparation and whole-genome sequencing. Sequence analysis revealed 4 unique mutations in PfActin1 (PF3D7_1246200) among the 29 confident missense mutations found in the core genome of Dd2-Pol δ. In the first biological replicate (flask 1), allele frequency estimates based on sequencing reads suggest that 24% of the resistant parasites possessed both A171S and I290L single-nucleotide variants (SNVs), and 76% harbored the SNVs of A171V and I290L (Fig. 2*A*). The same mutation (A136S) appeared in the second and third biological replicates (flasks 2 and 3).

Cloning by limiting dilution was then performed to isolate individual lines from the bulk population. Due to the multiple mutations identified in the first biological replicate, we isolated four clones from this bulk culture as compared to two clones each from the second and third replicates. These clones were phenotyped via a SYBR green I-based dose–response assay (Table 2 and Fig. 2*B*). An average resistance shift of 2.6-fold was verified in clones from flask 1, while those from flasks 2 and 3 had slightly higher EC_50_ shifts of 3.6 and 3.3, respectively (Table 2 and *SI Appendix*, Fig. S2 *A* and *B*). The DNA of these clones was then extracted and subjected to whole-genome sequencing. The sequencing results are summarized in Table 2.

**Table 2.** The resistance phenotypes of the in vitro evolved clones to NPDG-F and the cross-resistance analysis to cytochalasin D and jasplakinolide

							Cytochalasin D		Jasplakinolide	
Strain^*^	EC_50_(nM) (mean ± SEM)	Fold change	A136S^†^	A171S^†^	A171V^†^	I290L^†^	EC50 (nM) (mean ± SEM)	Fold change	EC50 (nM) (mean ± SEM)	Fold change
Dd2-Pol δ -parent	3,430 ± 433	−	−	−	−	−	17.3 ± 0.5	−	25.1 ± 1.4	−
1-C7	11,484 ± 164	3.3	−	Y (8%)	Y (92%)	Y	302.5 ± 8.8	17.5	25.2 ± 0.4	1.0
1-D6	7,791 ± 124	2.3	−	Y (3%)	Y (97%)	Y	317.4 ± 9.2	18.3	27.6 ± 4.0	1.1
1-D7	9,022 ± 243	2.6	−	Y (22%)	Y (78%)	Y	402.4 ± 7.0	23.3	25.9 ± 0.8	1.0
1-F3	7,035 ± 478	2.1	−	Y (4%)	Y (96%)	Y	366.9 ± 7.8	21.2	30.7 ± 3.2	1.2
2-C4	14,118 ± 591	4.1	Y				60.8 ± 0.8	3.5	22.4 ± 0.0	0.9
2-F5	10,211 ± 834	3.0	Y				71.3 ± 1.7	4.1	25.1 ± 2.9	1.0
3-C3	12,692 ± 826	3.7	Y				66.4 ± 0.2	3.8	24.0 ± 0.0	1.0
3-C5	10,042 ± 581	2.9	Y				73.2 ± 0.7	4.2	25.4 ± 2.1	1.0

^*^The number to the left of the hyphen indicates the flask from which the clone was isolated.

^†^The presence of the mutation was indicated as “Y” and otherwise it was left blank. The allele frequency was shown in parentheses; if not specified, it was 100%.

### Docking Study Elucidates the Relationship Between NPDG-F and the Binding Pocket of PfActin1.

Based on the mutations identified through the in vitro evolution of resistance, we set a docking grid around subdomains 1 and 3 of PfActin1 (PDB ID: 6TU4) (14). From this, we obtained a docking pose for NPDG-F with a docking score of -8.6 kcal/mol (Fig. 2 *C*–*E*). Four putative hydrogen bonds were predicted to fit NPDG-F into the hydrophobic cleft of subdomains 1 and 3 of PfActin1. Residues Y134, I137, G169, and Y144 were suggested to interact with the hydroxyl groups at carbon 20, 19, and 7 and the carbonyl group at carbon 23, respectively. In the same fashion, cytochalasin D was docked into PfActin1 6TU4 with a docking score of -5.4 kcal/mol (Fig. 2 *F*–*H*). Cytochalasin D docked to the same binding pocket as NPDG-F although only one hydrogen bond (with I137) was suggested by the docking (Fig. 2 *G* and *H*). We hypothesize that docking was not able to capture the conformational dynamics of the protein and therefore unable to reveal more than one hydrogen bond.

To investigate whether NPDG-F has the same mode of action as cytochalasin D, we tested our clones for sensitivity to both cytochalasin D and jasplakinolide. The clones were cross-resistant to cytochalasin D (Table 2 and Fig. 2*B* and *SI Appendix*, Fig. S2 *C* and *D*) but not to jasplakinolide (Table 2 and Fig. 2*B* and *SI Appendix*, Fig. S2 *E* and *F*). Clones from flask 1 had a 20-fold of EC_50_ shift while clones from flasks 2 and 3 had an average EC_50_ shift of 3.9-fold. These data corroborated results from the docking study, suggesting that cytochalasin D and NPDG-F share the same binding pocket while jasplakinolide binds to the opposite side of the protein (Fig. 2*C*).

Mutating the alanine to serine or valine at residue 171 rendered a 20-fold EC_50_ shift for cytochalasin D and a 2.6-fold shift for NPDG-F. As previously reported, cytochalasin D forms two hydrogen bonds with A170 of a *Drosophila melanogaster* actin (PDB No. 3EKS) (15), which corresponds to A171 of 6TU4 (*SI Appendix*, Fig. S3*A*). It has been shown that the hydrogen bonding involving the amide group of A171 was conserved in various barbed-end-targeting microlide toxins including cytochalasin D, bistramide A and jaspisamide A (15). It is thus possible that A171 forms hydrogen bond(s) with NPDG-F and cytochalasin D. The A171V/S mutation could affect the hydrogen bond formation, thereby causing significant potency loss.

The change in amino acid from alanine to serine at residue 136 resulted in a resistance shift of 3.9-fold for cytochalasin D and a comparable shift of 3.5-fold for NPDG-F. A previous study has shown that the mutation A136G confers resistance to cytochalasin D in *T. gondii* (16). The mechanism by which A136S confers resistance is unknown. However, we hypothesize that the change from a hydrophobic to a hydrophilic amino acid by the addition of one hydroxyl group may induce a conformational change in the protein, potentially affecting the hydrogen bonding at Y134 and I137 (Fig. 2 *D* and *E*). An alternative hypothesis is that the water molecule found near A136 (*SI Appendix*, Fig. S3*B*) could serve as a bridge, enabling the hydrogen bonding between the ligand and the protein, as is the case in cytochalasin D in 3EKS (*SI Appendix*, Fig. S3*A*). The A136S mutation could potentially disrupt the water-mediated hydrogen bonding.

### NPDG-F Behaves Antagonistically with Other Actin Interfering Drugs.

**Fig. 3.**
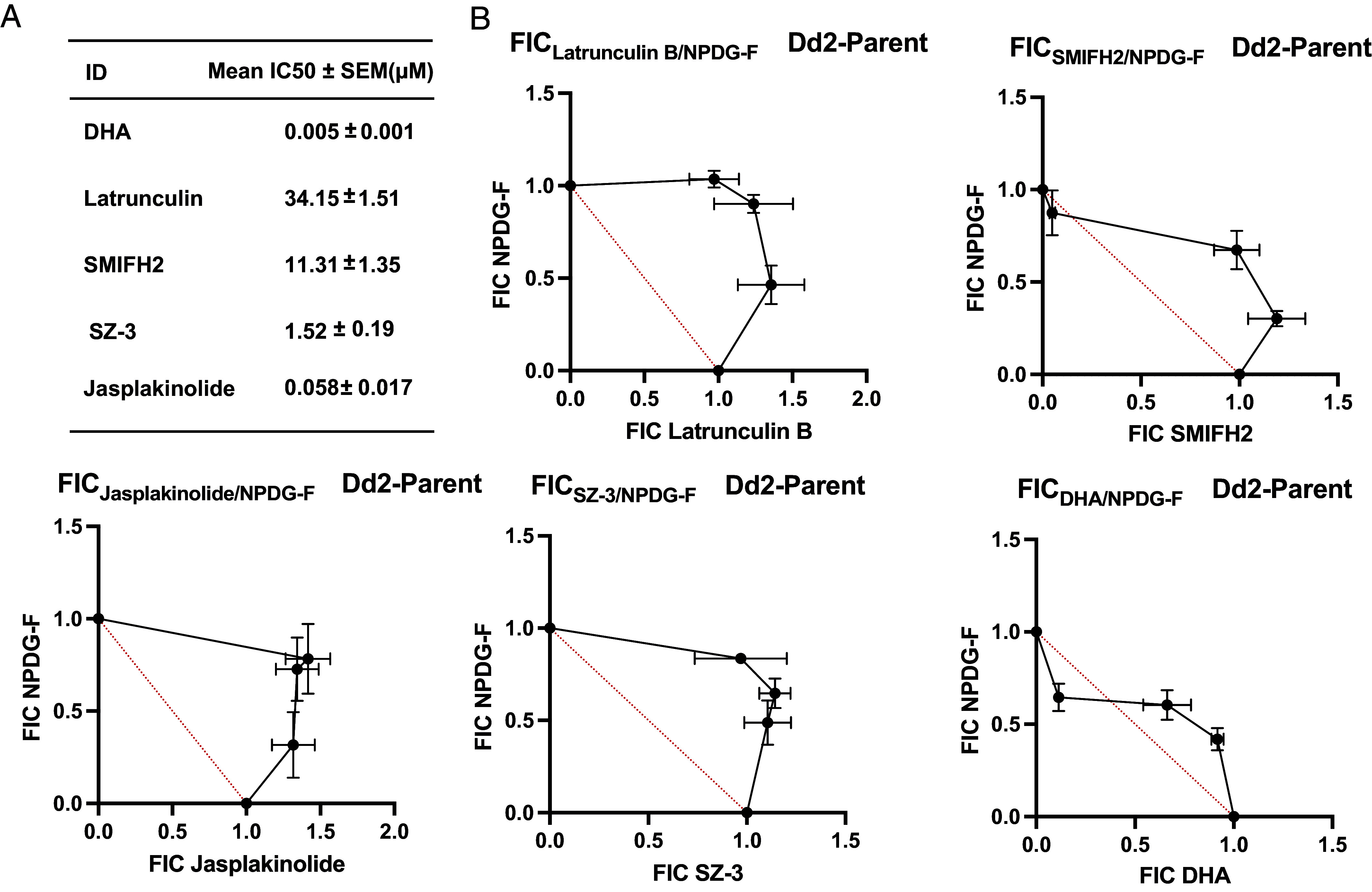
Methyldeoxaphomin NPDG-F isobolograms. (*A*) Dose–response EC_50_ determination for compounds tested in NPDG-F interaction study. (*B*) FIC interactions between NPDG-F and DHA (dihydroartemisinin) or other actin inhibitors including latrunculin B, SMIFH2, jasplakinolide, and SZ-3. Points above the curve suggest an antagonistic effect, while points below the curve suggest synergism. Red dotted lines indicate standard additive effect.

To further explore the relationship of NPDG-F with established actin-interfering drugs, an isobologram study was performed (Fig. 3). NPDG-F was added in combination with four inhibtors known to interfere with actin polymerization including a formin inhibitor SMIFH2, cofilin inhibitor SZ-3, F-actin targeting compound jasplakinolide, and G-actin targeting latrunculin B. The frontline antimalarial DHA was also added as a nonactin inhibitor control. After dose–response curves were generated (Fig. 3*A*), the fractional inhibitory concentrations (FICs) of NPDG-F and the combination drug were calculated and graphed. As seen in Fig. 3*B*, all four of the actin-interfering test inhibitors showed an antagonistic relationship with NPDG-F, meaning higher than expected concentrations of each inhibitor were required to produce the desired effect. The majority of the FIC’s of the actin-targeting inhibitors tended to cluster toward one extremity (>1), with jasplakinolide and latrunculin B showing the highest FIC values overall. SMIFH2 however, was primarily indifferent to the addition of NPDG-F at very low concentrations, and antagonistic at moderate to high concentrations. Interestingly, the nonactin inhibitor DHA was found to be somewhat synergistic with NPDG-F at low concentrations, and antagonistic at higher concentrations.

### The Stage-Specific Activity of the NPDG-F Identifies Abnormalities in Merozoite Segmentation.

**Fig. 4.**
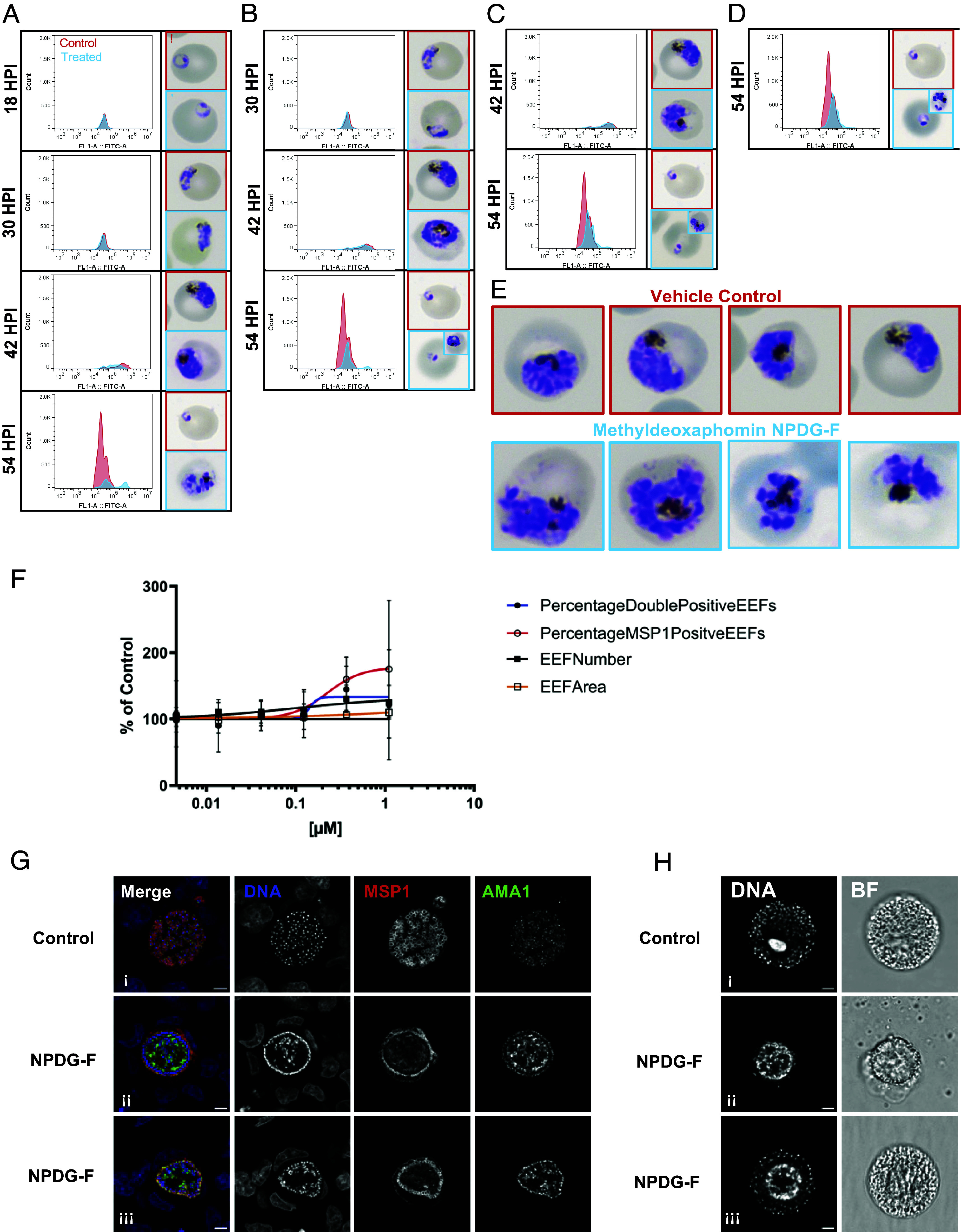
Stage-specific activity of methyldeoxaphomin NPDG-F. A synchronous Dd2 culture was incubated with 5 × EC_50_ of methyldeoxaphomin NPDG-F or a DMSO vehicle control at select timepoints of (*A*) 6, (*B*) 18, (*C*) 30, or (*D*) 42 h post synchronization (HPS). Samples were then taken every 12 h until 54 HPS for flow cytometric analysis with YOYO-1 and Giemsa stain thin smear. The control flow and Giemsa images are indicated in red, with treated samples in blue. Results representative of three biological replicates are shown. (*E*) Giemsa staining highlighting abnormal segregation during schizogony. (*F*) HepG2 cells were infected with *P. berghei* sporozoites and treated with NPDG-F. Readouts were taken at 72 HPI. EEF: exoerythrocytic form. Percentages of MSP1 positive and double (MSP1 and AMA1) positive parasite were graphed. (*G*) Representative, single confocal images of *P. berghei* infected HepG2 treated with 1.1 µM of NPDG-F or DMSO control, fixed at 55 HPI. Single-channel images were shown in grayscale and merged images were shown in color. DNA was stained with Hoechst (blue), MSP1 signal was shown in red, and AMA1 in green (Scale bar, 5 µm). (*H*) Images from live merosome imaging taken at 72 HPI. Full videos are available in Movies S1 and S2 (Scale bar = 5 µm).

To corroborate the findings identified through the in vitro evolution of resistance, the activity of NPDG-F was assessed in a stage-dependent manner in vitro. Because actin proteins are needed for chromosome separation, we anticipated that parasites treated with NPDG-F would arrest late in the cell cycle. The parasite strain Dd2 was first synchronized and treated in ring (~6 h post invasion, HPI), trophozoite (~18 HPI), early schizont (~30 HPI), or late schizont (~42 HPI). Cultures were then observed in each condition every 12 h until reinvasion occurred via Giemsa stain and flow cytometry. When added in early ring, NPDG-F treated cultures proceeded normally through the intraerythrocytic life cycle until late schizogony (Fig. 4*A*). At schizogony, parasites showed numerous irregularities in merozoite segmentation and complete life cycle inhibition. When NPDG-F was added after 6 HPI however, this phenotype persisted only in a minor population of schizonts (Fig. 4 *B*–*D*). Parasitemia during the second life cycle was markedly lower than in control populations, and the majority of parasites observed were able to reinvade and proceed into early ring. Taken together, these results suggest that NPDG-F affects schizogony with a phenotype of impaired merozoite separation (Fig. 4*E*) and an extended incubation time is needed for complete growth inhibition. These findings are in line with the gradual killing rate seen in NPDG-F treated cultures, and the extended lag phase of 24 h.

### NPDG-F Treatment Impedes Cellularization During the Late Stage of Schizogony in the Liver Stage of *Plasmodium berghei*.

Given these findings, we sought to observe the effects of NPDG-F on hepatic merozoites to see whether they were similarly impacted. To this end, HepG2 cells were infected with *P. berghei* sporozoites expressing luciferase and seeded onto a microtiter plate with NPDG-F at 2 HPI. At 72 HPI, a high content imaging (HCI) readout of treated parasites was performed to observe the impact on parasite maturation and merozoite formation via the expression of liver stage maturation markers MSP1 and AMA1. Interestingly, NPDG-F caused a sharp increase in MSP1 positive exoerythrocytic form (EEF) parasites remaining in the monolayer, although this increase varied noticeably in magnitude between replicates, suggesting that late liver stage maturation was affected (Fig. 4*F*). Infected monolayers treated with 1.1 µM of methyldeoxaphomin NPDG-F from 2-55 HPI were immunolabeled to examine the localization of MSP1 and AMA1 relative to parasite DNA during the period of merozoite formation (Fig. 4*G*). NPDG-F treatment of the parasite resulted in developmental failure during schizogony, with DNA largely clumped in the middle of the schizont, instead of being distributed to each individual merozoite, with MSP1 and AMA1 robustly expressed, but mislocalized (Fig. 4*G*), while control merozoites show MSP and AMA1 surrounding the nucleus (Fig. 4 *G, i*). As both MSP1 and AMA1 are localized to the plasma membrane surrounding each merozoite, while the NPDG F-treated parasites show accumulations of MSP1 and AMA in the undifferentiated center of the parasite as well as at the very edge (Fig. 4 *G, ii, iii*). The phenotype varied in severity, with some parasites showing limited merozoite formation with seemingly aberrant AMA1 localization (Fig. 4 *G, iii*), while others seemed to fail completely at merozoite formation (Fig. 4 *G, ii*) Live imaging of merosomes taken at 72 HPI (Fig. 4*H* and Movies S1 and S2) reinforced this phenotype distinction. In summary, NPDG-F treatment of the liver-stage parasites rendered a sharp reduction in merozoite cellularization which was in line with results found in the blood stage (Fig. 4*E*). Taken together, the developmental defects induced by NPDG-F in both blood and liver-stage schizonts are consistent with a previous study showing that PfActin1 deficiency results in the failure of daughter cell separation during blood-stage schizogony (17).

## Discussion

Although actin proteins are highly conserved across eukaryotes, the two actin isoforms of *P. falciparum* are divergent sharing less than 80% sequence similarity with each other and with mammalian actins (18). In addition to their structural and functional differences, apicomplexan actins display unique dynamics. Despite *T. gondii* actin possessing an isodesmic polymerization (19), in *P. falciparum*, polymerization is accomplished through the traditional nucleation-elongation process with a critical concentration (4 µM) being an order of magnitude higher than that of the canonical mammalian actin (20). However, another study has shown that the critical concentration is around 0.1 μM using pyrene-labeled PfActin1 and fluorescence spectroscopy in conjunction with dynamic light scattering (21). PfActin1 and PfActin2 were both shown to hydrolyze ATP more efficiently than alpha-actin, and both rapidly oligomerize in the presence of ADP, which is distinct from traditional actins (18). Structurally, *PfActin1* differs greatly from canonical actin, forming unstable, short filaments (~100 nm) (18). The short length of the filament has been associated with fast actin turnover (21). These structural and dynamic differences between mammalian and *Plasmodium* actins offer promise for target-based drug discovery efforts to search for new therapeutics. In a recent study, the small-molecule MMV020291 was found to target PfActin1 and profilin, leading to failure in actin filament formation in vitro and apicoplast segregation in vivo (22).

The role of PfActin1 in parasite growth and development has been shown in conditional deletion studies (17). Despite the normal formation of micronemes and normal secretion, PfActin1 deficiency resulted in a failure of both apicoplast migration to individual merozoites and daughter cell separation at the final stage of cytokinesis. These merozoites were frequently found to be conjoined and still attached to food vacuole and incapable of invading new host cells (17). Similar defects were observed in the *P. berghei* liver stage parasites treated with NPDG-F, suggesting arrested growth at the schizont stage due to cellularization failure, a hallmark of actin deficiency. The observed cellularization failure in our study could be due to delays in schizogony or abnormalities in the budding process causing disruptions in the final round of semisynchronous nuclear division and daughter cell formation (23).

The docking study identified key residues in the binding pocket of NPDG-F in both human skeletal alpha actin (PDB ID: 6VAO) (24) and PfActin1, which may suggest a challenge in developing selective inhibitors due to the conserved nature of these residues (*SI Appendix*, Fig. S3*C*). However, with a HepG2 cell EC_50_ of >25 μM and a selectivity of >60 (Table 1), NPDG-F shows promise as a PfActin1-targeted lead compound. The sequence conservation in the binding pockets of human and *Plasmodium* does not necessarily mean that an inhibitor that was specific for PfActin1 could not be created. The fungus developed NPDG-F as a defense mechanism against a myriad of other organisms, which required it to be broadly effective rather than highly specific: *T. asperum* is probably not infected by malaria parasites, naturally. In sequencing of the *T. asperum* genome, we found that alanine was replaced with serine at residue 136 (*SI Appendix*, Fig. S3*C*). This highlights the widespread occurrence and significance of the A136S mutation in conferring resistance to NPDG-F, both in the natural environment and laboratory setting.

Cytochalasin D (CD) inhibits actin dynamics by binding to the barbed end of F-actin with a stoichiometry of one molecule per filament (25). Cytochalasin D impacts actin dynamics through multiple mechanisms. Co-crystallization of cytochalasin D and *Drosophila melanogaster* actin resulted in the ~6° rotation of small subdomains 1 and 2 relative to the larger subdomains 3 and 4 (15). This caused subdomain 1 to twist backward and subdomain 2 to twist forward, which in turn influenced monomer packing and the conformation of the D-loop. The otherwise disordered D-loop took on an extended and ordered conformation stabilized by two hydrogen bonds with a neighboring actin monomer which then “buried” cytochalasin D in its binding pocket (15). Our docking study also suggests that the D-loop (residues 40-50) extended toward subdomains 1 and 3 of the adjacent actin monomer along the filament (*SI Appendix*, Fig. S3*D*). The hydrophobic pocket of CD binding is a hotspot for numerous actin-binding proteins including cofilin, profilin, and gelsolin (26, 27). By binding to the hydrophobic cleft, CD prevents other proteins from accessing the cleft. Cytochalasin D was shown to inhibit the binding of cofilin to G- and F-actin (25) thus affecting cofilin-mediated actin turnover. Owing to the small size of CD and its high binding affinity (K_d_ ≈ 2 nM), CD thus “caps” the filament and affects monomer association and dissociation from this end (15). NPDG-F likely acts similarly to CD based on the docking studies and the cross-resistance observed in the *in vitro-*evolved resistant lines.

In conclusion, the work presented here established the antiplasmodial activity of methyldeoxaphomin NPDG-F, an inhibitor that likely targets PfActin1. This finding is supported by microscopy in both blood and liver stages, antagonism seen between NPDG-F and known actin-binding inhibitors, the cross-resistance of in vitro evolved lines to the known actin inhibitor cytochalasin D, and docking study. Further molecular dynamics studies could help to augment the docking study as they suffer from limitations due to their inability to capture the conformational dynamics of the protein–ligand interaction. Co-crystallization of PfActin1 with NPDG-F could help elucidate the chemical interactions between the ligand and the receptor. Additionally, genetic editing via CRISPR/Cas9 to introduce the mutations found through in vitro evolution would further validate PfActin1 as the putative target. Nonetheless, the data presented here elucidate the in vitro activity of NPDG-F in both the plasmodial blood and liver stages and highlights its ability to act as an antiplasmodial agent through the inhibition of PfActin1.

## Materials and Methods

Comprehensive details of the experimental methods, reagents, and equipment are provided in *SI Appendix*. The *Materials and Methods* are organized according to the workflow, beginning with compound isolation and purification, followed by antimalarial characterization of the most potent compound, and concluding with drug target identification. In the chemistry part, we detailed procedures including fungal material and fermentation, X-ray crystallographic analysis, and structural elucidation of metabolite 1(NPDG-A). For the antimalarial characterization of methyldeoxaphomin NPDG-F, we detailed the experiments including isobologram assay, stage-specific assay, and parasite reduction ratio. For the target identification and molecular mechanism studies, we recorded methods for in vitro evolution of resistance, whole genome sequencing analysis, high content imaging,3D structural analysis of the mutations and docking study.

## Supplementary Material

Appendix 01 (PDF)

Dataset S01 (XLSX)

Dataset S02 (XLSX)

Dataset S03 (XLSX)

Movie S1. Live imaging of merosomes taken at 72 HPI. This is a video representation of Figure 4H(ii), showing the strong inhibition of merozoite formation. Scale bars = 5µm

Movie S2. Live imaging of merosomes taken at 72 HPI. This is a video representation of Figure 4H(iii), showing the weak inhibition of merozoite formation. Scale bars = 5µm

## Data Availability

Whole genome sequencing data of *Trichocladium asperum*, one parent (Dd2- Pol δ) and 8 in vitro evolved clones of *Plasmodium falciparum* data have been deposited in Sequence Read Archive (SRA) (No. PRJNA1112907) (28). All study data are included in the article and/or supporting information.
