# Supplementary material for: Fungal-derived methyldeoxaphomins target Plasmodium falciparum segregation through the inhibition of PfActin1
Source: Proc Natl Acad Sci U S A. 2025 Feb 18;122(8):e2418871122. doi: 10.1073/pnas.2418871122 (PMC11874377; doi:10.1073/pnas.2418871122)
Supplement: Supplementary file 1 — Appendix 01 (PDF) [file pnas.2418871122.sapp.pdf]

PNAS

Supporting Information for

Fungal Derived Methyldeoxaphomins Target *Plasmodium falciparum* Segregation Through the Inhibition of PfActin1

Tiantian Jiang[a]#, Jin Woo Lee[b]#, Jennifer E. Collins[c] #, Samuel Schaefer[a], Daisy Chen[a], Flore Nardella[c], Karen Wendt[d], Thilini G. Peramuna[d], Raphaella Paes[c], James L. McLellan[e], Jasveen Bhasin[c] Gregory L. Durst[f], Kirsten K. Hanson[e], Debopam Chakrabarti[c], Robert H. Cichewicz[d], Elizabeth A. Winzeler[a,g]*

#Equal contribution.

[a] Department of Pediatrics, School of Medicine, University of California, San Diego, La Jolla, CA, 92093, United States.

[b] College of Pharmacy, Duksung Women's University, Seoul, 01369, Republic of Korea.

[c] Division of Molecular Microbiology, Burnett School of Biomedical Sciences, University of Central Florida, 12722 Research Parkway, Orlando, Florida 32826, United States.

[d] Department of Medicinal Chemistry, College of Pharmacy, University of Michigan, Ann Arbor, Michigan, 48109, United States.

[e]Department of Molecular Microbiology and Immunology, South Texas Center for Emerging Infectious Diseases, University of Texas at San Antonio, San Antonio, Texas, 78249, United States

[f]Lgenia Inc., 412 S Maple St - Suite 104, Fortville, Indiana, 46040, United States

[g]Skaggs School of Pharmacy and Pharmaceutical Sciences, University of California, San Diego, La Jolla, CA, 92093, United States

Corresponding Authors

*E-mail: ewinzeler@health.ucsd.edu; rcic@umich.edu; dchak@ucf.edu

This PDF file includes:

General experimental procedures
Fungal material and fermentation
Extraction and isolation
X-ray crystallographic analysis of metabolite 1
Structure elucidation of novel methyldeoxaphomin analogs
Plasmodium blood stage culture
Antiplasmodial activity screening
Isobologram assay
Cytotoxicity screening
Stage specific activity assay
Rate of killing assay
Parasite reduction ratio
High content imaging liver stage screening
Plasmodium in vitro evolution of resistance
Whole genome sequencing analysis
Limiting dilution to generate clonal lines
Resistant parasite phenotyping and cross resistance measurement
3D structural analysis of the mutations and docking studies
Figure S1. Killing rate profile of methyldeoxaphomin NPDG-F
Figure S2. Phenotyping of the resistant clones and the cross-resistance tests to cytochalasin D and jasplakinolide
Figure S3. High similarity of the binding pockets of NPDG-F with human and *Plasmodium* actins
Table S1. ^{1}H (500 MHz) and ^{13}C NMR (100 MHz) data of compounds 1-3 (NPDG -A to C) in $CDCl_3$
Table S2. ^{1}H (500 MHz) and ^{13}C NMR (100 MHz) data of compounds 4-6(NPDG-D to F) in $CDCl_3$
SI References

Other supporting materials for this manuscript include the following:

Movie S1(separate file). Live imaging of merosomes taken at 72 HPI. This is a video representation of Figure 4H(ii), showing the strong inhibition of merozoite formation. Scale bars = 5µm

Movie S2(separate file). Live imaging of merosomes taken at 72 HPI. This is a video representation of Figure 4H(iii), showing the weak inhibition of merozoite formation. Scale bars = 5µm

Dataset S1(separate file). The antimalarial activities of the six compounds and their cytotoxicity

Dataset S2(separate file). The copy number variant (CNVs) analysis of the parent line and the resistant clones

Dataset S3(separate file). The single nucleotide variant analysis (SNVs) of the parent line and the resistant clones

General experimental procedures

Optical rotations were measured on a Rudolph Research Autopol III automatic polarimeter. NMR spectra were obtained on Varian 400 and 500 MHz NMR spectrometers. HRESIMS data were measured on an Agilent 6538 high-mass-resolution QTOF mass spectrometer. ECD spectra were obtained on a JASCO J-715 circular dichroism spectrometer. Column chromatography was performed on silica gel (VWR, 40-60 μm, 6 Å) and HP20ss gel (Sorbtech). The preparative HPLC system was equipped with SCL-10A VP pumps and system controller and a Gemini 5 μm C_{18} column (110 Å, 250 × 21.2 mm). The semi-preparative HPLC were conducted on Waters system (1525 binary pumps and Waters 2998 photodiode array detectors) using Gemini 5 μm C_{18} columns (110 Å, 250 × 10 mm) and F5 5 μm columns (100 Å, 250 × 10 mm). All solvents were of ACS grade or better.

Fungal material and fermentation

The *Trichocladium* sp. isolate (TN09213 RBM-1) was acquired from a soil sample collected near Lebanon, Tennessee, USA by a citizen scientist. The fungus was grown on a Petri plate and fresh mycelium was collected and subjected to homogenization in TE buffer (10 mM EDTA HCl, 0.1 mM EDTA, pH 8.0) with zirconium oxide beads in a Bullet blender (MidSci #BBY24M). Fungal DNA was collected, and the ITS region (i.e., ITS1, 5.8S, and ITS2 regions) was amplified by PCR and sent for sequencing. The sequence was compared to fungal sequences stored in GenBank, which led to two hits with 100% identity to those from *Trichocladium asperum* and *Trichocladium griseum*. Sequence data were deposited in GenBank (*T. asperum*: GenBank accession no. MZ230232). Subsequently, to obtain fungal extracts, the fungus was grown on Cheerios breakfast cereal supplemented with a 0.3% sucrose solution and 0.005% chloramphenicol in three large mycobags (Unicorn Bags, Plano, TX, USA) for 4 weeks

Extraction and isolation

The fungal cultures were extracted with EtOAc (3 × 2L) at room temperature to yield the EtOAc extract of 33 g (Fraction A). Fraction A was chromatographed by silica gel vacuum column chromatography obtaining fraction B (Dichloromethane 100%), fraction C (Dichloromethane:MeOH = 10:1) and fraction D (MeOH 100%). Fraction C (6.0 g) was separated into three sub-fractions, fractions C70 (70% MeOH), C90 (90% MeOH) and C100 (100% MeOH), by HP20ss gel vacuum column chromatography. Fraction C90 (300 mg) was further subjected to preparative HPLC (C_{18}, 70 to 100% MeOH/H_2O in 15 min, flow rate: 10 mL/min) getting thirteen subfractions (C90A~M). Among the subfractions C90A~M, C90F and C90H were identified to be pure compounds, **5** (15 mg) and **2** (10 mg), respectively. Subfraction C90B (30 mg) was applied on semi-preparative HPLC (C_{18}, ACN:Water = 45:55, flow rate: 4 mL/min) to yield compound **6** (3 mg). Compound **3** (6 mg) was purified from subfraction C90C (20 mg) by semi-preparative HPLC (C_{18}, ACN:Water = 60:40, flow rate: 4 mL/min). Subfraction C90J (25 mg) was subjected to semi-preparative HPLC (C_{18}, ACN:Water = 60:40, flow rate: 4 mL/min) to afford **1** (7 mg) and **4** (2 mg). Fraction D (5.0 g) was also separated into three sub-fractions, fractions D70 (70% MeOH), D90 (90% MeOH) and D100 (100% MeOH), by HP20ss gel vacuum column chromatography. Fraction D90 (1 g) was further subjected to preparative HPLC (C_{18}, 70 to 100% MeOH/H_2O in 15 min, flow rate: 10 mL/min) to obtain ten subfractions (D90A~J). Among the subfractions D90A~J, D90C, D, E, G and H were turned out to be single compounds determined as compounds **7** (13 mg), **8** (11 mg), **9** (9 mg), **10** (12 mg) and **11** (14 mg), respectively.

Methyldeoxaphomin NPDG-A (**1**): white powder; $[\alpha]_D^{25}$ +46 (*c* 0.1, MeOH); UV (MeOH) λ_{max} (log ε) 200 (3.3), 218 sh, 242 (1.9) nm; ECD (*c* 0.01 MeOH) λ_{max} (mdeg) 214 (-27.7), 251 (-6.1), 336 (+1.5); ^{1}H NMR (500 MHz, $CDCl_3$) and ^{13}C NMR (100 MHz, $CDCl_3$), see Table 1; HRESIMS *m/z* 498.2624 $[M+Na]^+$ (calcd for $C_{30}H_{37}NNaO_4$, 498.2615; mass error +1.8 ppm).

Methyldeoxaphomin NPDG-B (**2**): yellow powder; $[\alpha]_D^{25}$ +40 (*c* 0.1, MeOH); UV (MeOH) λ_{max} (log ε) 200 (2.5), 218 sh, 242 (1.7) nm; ECD (*c* 0.01 MeOH) λ_{max} (mdeg) 214 (-51.1), 253 (-7.8), 340 (+2.9); ^{1}H NMR (500 MHz, $CDCl_3$) and ^{13}C NMR (100 MHz, $CDCl_3$), see

Table 1; HRESIMS *m/z* 514.2572 $[M+Na]^+$ (calcd for $C_{30}H_{37}NNaO_5$, 514.2564; mass error +1.6 ppm).

Methyldeoxaphomin NPDG-C (**3**): white powder; $[\alpha]_D^{25}$ +58 (*c* 0.1, MeOH); UV (MeOH) λ_{max} (log ε) 200 (2.7), 218 sh, 242 (2.2) nm; ECD (*c* 0.01 MeOH) λ_{max} (mdeg) 209 (-21.0), 226 (+3.4), 308 (+1.9); ^1H NMR (500 MHz, $CDCl_3$) and ^{13}C NMR (100 MHz, $CDCl_3$), see Table 1; HRESIMS *m/z* 514.2574 $[M+Na]^+$ (calcd for $C_{30}H_{37}NNaO_5$, 514.2564; mass error +1.9 ppm).

Methyldeoxaphomin NPDG-D (**4**): white powder; $[\alpha]_D^{25}$ -40 (*c* 0.1, MeOH); UV (MeOH) λ_{max} (log ε) 200 (2.3), 218 sh, 242 (1.5) nm; ECD (*c* 0.01 MeOH) λ_{max} (mdeg) 218 (-6.0), 253 (-1.5), 341 (+0.5); ^1H NMR (500 MHz, $CDCl_3$) and ^{13}C NMR (100 MHz, $CDCl_3$), see Table 2; HRESIMS *m/z* 514.2570 $[M+Na]^+$ (calcd for $C_{30}H_{37}NNaO_5$, 514.2564; mass error +1.2 ppm).

Methyldeoxaphomin NPDG-E (**5**): red gum; $[\alpha]_D^{25}$ +66 (*c* 0.1, MeOH); UV (MeOH) λ_{max} (log ε) 200 (3.0), 218 sh, 242 (2.3) nm; ECD (*c* 0.01 MeOH) λ_{max} (mdeg) 212 (-17.3); ^1H NMR (500 MHz, $CDCl_3$) and ^{13}C NMR (100 MHz, $CDCl_3$), see Table 2; HRESIMS *m/z* 500.2778 $[M+Na]^+$ (calcd for $C_{30}H_{39}NNaO_4$, 500.2771; mass error +1.4 ppm).

Methyldeoxaphomin NPDG-F (**6**): white powder; $[\alpha]_D^{25}$ +48 (*c* 0.1, MeOH); UV (MeOH) λ_{max} (log ε) 200 (3.7), 218 sh, 242 (3.0) nm; ECD (*c* 0.01 MeOH) λ_{max} (mdeg) 211 (-31.5), 255 (+4.3), 324 (+1.2); ^1H NMR (500 MHz, $CDCl_3$) and ^{13}C NMR (100 MHz, $CDCl_3$), see Table 2; HRESIMS *m/z* 516.2720 $[M+Na]^+$ (calcd for $C_{30}H_{39}NNaO_5$, 516.2730; mass error +1.9 ppm).

X-ray crystallographic analysis of metabolite 1

Crystals of compound **1** were obtained from MeOH. A yellow, needle-shaped crystal of dimensions 0.074 × 0.074 × 0.404 mm was selected for structural analysis. Intensity data were collected using a D8 Quest *κ*-geometry diffractometer with a Bruker Photon II cmos

area detector and an Incoatec Iμs microfocus Mo K α source (λ = 0.71073 Å). The sample was cooled to 101(2) K. Cell parameters were determined from a least-squares fit of 9863 peaks in the range 2.44 < θ < 26.27°. A total of 47648 data were measured in the range 2.443 < θ < 26.441° using ϕ and ω oscillation frames. The data were corrected for absorption by the empirical method giving minimum and maximum transmission factors of 0.6838 and 0.7454. The data were merged to form a set of 5294 independent data with *R*(int) = 0.0956 and a coverage of 99.9%. Crystallographic data for **1** have been deposited at the Cambridge Crystallographic Data Centre (deposition number: CCDC 2337144). Copies of these data can be obtained free of charge from the CCDC via www.ccdc.cam.ac.uk.

Structure elucidation of novel methyldeoxaphomin analogs

Metabolite **1** was obtained as a white amorphous powder, and its molecular formula was determined as $C_{30}H_{37}NO_4$ by the HRESIMS spectrum (*m/z* 498.2624 $[M+Na]^+$; calcd for 498.2615). Interpretation of the 1D NMR (^{1}H and ^{13}C) and the HSQC spectra supported that **1** contained two ketone groups (δ_C 203.16, 195.74), an amide group [δ_H 5.54 (N**H**); δ_C 171.50], a benzyl group [δ_H 7.32 (m, 2H), 7.14 (m, 2H), 7.26 (m), 3.01 (dd, *J* = 14.0, 3.5 Hz), 2.48 (dd, *J* = 14.0, 10.0 Hz); δ_C 137.12, 129.04 ´ 2, 128.94 ´ 2, 127.20, 44.67), an *exo*-methylene [δ_H 5.47 (br t, *J* = 2.0 Hz), 5.19 (br t, *J* = 2.0 Hz); δ_C 148.55, 112.84], two *trans*-double bonds [δ_H 7.81 (d, *J* = 16.5 Hz), 6.73 (d, *J* = 16.5 Hz), 5.96 (dd, *J* = 15.0, 10.0 Hz), 5.83 (dd, *J* = 15.0, 8.5, 6.0 Hz); δ_C 135.27, 173.31, 129.04, 137.33], an oxymethines [δ_H 4.03 (dd, *J* = 10.0, 2.0 Hz); δ_C 69.43] and three secondary methyls [δ_H 1.26 (d, *J* = 7.0 Hz), 1.00 (d, *J* = 7.0 Hz), 0.90 (d, *J* = 6.5 Hz); δ_C 15.75, 23.75, 21.37] (**Table S1**). Analysis of the ^{1}H-^{1}H COSY spectrum gave two major C-linkage fragments, C-7,8/C-13,14,15,16(24),17,18(25),19 and C-10/C-3,4,5/C-11, as shown in bold line in **Figure 1A** The aforementioned moieties as well as the two big partial structures were completely assembled by the following HMBC correlations.

First, the HMBC correlations from H-12 to C-5,6,7 and from H-8 to C-1,4,9 indicated that the formation of a six-membered carbon ring connecting the two major partial structures

by *exo*-methylene and C-9 (**Figure 1A**). Second, the HMBC correlations of H-2',6' and C-10, H-9,3 and C-1, and NH and C-3, 9 suggested that the g-lactam ring was attached adjacent to the previously mentioned six-membered ring, and, furthermore, the benzyl moiety was positioned at C-3 in the lactam ring. Third, the position of one ketone was established by the HMBC correlations between H-18,19,21,22 and C-20. Finally, the HMBC correlations from H-4,8,21,22 to another ketone C-23 completely elucidated the planar structure of **1** by determining the formation of another macrocyclic ring. The structure of **1** closely resembled the known deoxaphomin analogue, a member of the cytochalasin family, which have been reported to exhibit various bioactivities, with a notable distinction being the presence of an additional methyl group at C-18 (Chen et al., 2015; Hu et al., 2022; Kim et al., 2012; Robert & Tamm, 1975). The 18-Methyldeoxaphomin is a highly rare scaffold, first reported in 2023, containing only three analogues (Meng et al., 2022).

The relative configurations of the isoindole core in **1** turned out to be the same as most of deoxaphomin and cytochalasin analogues based on the key ROESY correlations of H-10/H-4, H-4/H-5, H-5/H-8 and H-3/H-11 (**Figure 1B**). The configuration of C-9 could not be elucidated by the ROESY spectrum because of the absence of a proton, so it was determined based on biosynthetic considerations. However, even though the relative configurations of C-16 and C-18 were presumed by the ROESY experiment in several papers, determining them was expected to be challenging due to those chiral carbon connected by single bonds in the macrocyclic ring. After multiple attempts, therefore, the crystal of **1** was successfully obtained using MeOH and subjected to single-crystal X-ray diffraction analysis, which served to elucidate the relative configuration of the entire structure including C-16 and C-18 (**Figure 1D**). According to the X-ray analysis results, the configuration of C-18 of the methyldeoxaphomin series was uniquely revealed to differ from that of previously reported deoxaphomin analogues. The absolute configuration of **1** was determined by comparing its ECD spectrum with those f deoxaphomin and cytochalasin derivatives. Based on the negative Cotton effects at 214 and 251 nm and the positive Cotton at 336 nm, the absolute structure of **1** was established as 13*E*,21*E*-

3*S*,4*R*,5*S*,7*S*,8*R*,9*R*,16*S*,18*S*-18-methyl-20-oxo-deoxaphomin, and it was given the trivial name methyldeoxaphomin NPDG-A (**1**) (**Figure 1C**).

Metabolite **2** was obtained as a yellow powder, and assigned as $C_{30}H_{37}NO_5$ by the HRESIMS spectrum 514.2572 $[M+Na]^+$ (calcd for 514.2564). Interpretation of 1D and 2D NMR spectrum suggested that the structure of **2** was similar to methyldeoxaphomin NPDG-A (**1**) except for the presence of a hydroxy group at C-19 instead of the methylene group (δ_H 5.01, δ_C 74.51 in **2**; δ_H 3.24 and 1.93, δ_C 46.82 in **1**) (**Table S1 and Figure 1A**). The ROESY correlations in the isoindole core of **2** were identical to those of **1**, and the configurations of C-16 and 18 were determined based on the biosynthesis within the same species (JBCA and Mosher's method) (**Figure 1B**). Based on the negative Cotton effects at 214 and 253 nm and the positive Cotton at 340 nm, the absolute structure of **2** was established as 13*E*,21*E*-3*S*,4*R*,5*S*,7*S*,8*R*,9*R*,16*R*,18*S*,19*S*-18-methyl-19-hydroxy-20-oxo-deoxaphomin, and it was given the trivial name methyldeoxaphomin NPDG-B (**2**) (**Figure 1C**).

Metabolite **3** was purified as a white powder, and it was assigned the molecular formula $C_{30}H_{37}NO_5$ based on the analysis of the HRESIMS data 514.2574 $[M+Na]^+$ (calcd for 514.2564). The 1D and 2D NMR data for **3** indicated that the structure of **3** was similar to methyldeoxaphomin NPDG-B (**2**) (**Table S1 and Figure 1A**). The key difference between the metabolites was the (*E*)-configuration double bond at C-21 and C-22 in **2** had changed to a (*Z*)-configuration in **3**. The ROESY correlations in the isoindole core of **3** were identical to those of **1** and **2**, and, notably, the ROESY correlation of H-4 and H-22 were observed, allowing for the confirmation of the relative configuration of C-9 (JBCA and Mosher's method) (**Figure 1B**). Based on the negative Cotton effects at 209 nm and the positive Cotton at 226 and 308 nm, the absolute structure of **3** was established as 13*E*,21*Z*-3*S*,4*R*,5*S*,7*S*,8*R*,9*R*,16*R*,18*S*,19*S*-18-methyl-19-hydroxy-20-oxo-deoxaphomin, and it was given the trivial name methyldeoxaphomin NPDG-C (**3**) (**Figure 1C**).

Metabolite **4** was isolated as a white powder, and its molecular formula was determined to be $C_{30}H_{37}NO_5$ 514.2574 $[M+Na]^+$ (calcd for 514.2564), based on interpretation of the

HRESIMS data. Examination of the 1D and 2D NMR data for **4** revealed that the structure of **4** shared many structural features with methyldeoxaphomin NPDG-B (**2**), however, the key difference was shown in the isoindole part (**Table S2 and Figure 1A**). The olefinic bond of *exo*-methylene and the hydroxy group at C-7 had shifted to C-6 and C-7 inside the isoindole and C-12, respectively, generating an olefinic proton (δ_H 5.60) and a hydroxymethyl group at C-6. The analysis of the ROESY spectrum for **4** revealed the same relative configurations of the isoindole moiety as methyldeoxaphomin NPDG-A to C (**1**-**3**) (JBCA and Mosher's method) (**Figure 1B**). Based on the negative Cotton effects at 218 and 253 nm and the positive Cotton at 341 nm, the absolute configuration of **4** was established as 13*E*,21*E*-3*S*,4*R*,5*S*,8*S*,9*S*,16*R*,18*S,*19*S*, and it was given the trivial name methyldeoxaphomin NPDG-D (**4**) (**Figure 1C**).

Metabolite **5** appeared as a red gum, and its molecular formula ($C_{30}H_{39}NO_4$) was assigned based on interpretation of its HRESIMS data (*m/z* 500.2778 $[M+Na]^+$, calcd for $C_{30}H_{39}NNaO_4$, 500.2771). Considering the metabolite's molecular formular, as well as comparing its 1D and 2D NMR data with those of **1**, we had determined that the structure of **5** represents the reduced form of the ketone at C-20 in **1**, resulting in a hydroxy group (**Table S2 and Figure 1A**). The relative configuration of **5** was identical to those of compounds **1**-**4**, which was supported by the ROESY experiment and biosynthetic consideration within species (JBCA and Mosher's method) (**Figure 1B**). Based on the negative Cotton effects at 212 nm, the absolute structure of **5** was established as 13*E*,21*E*-3*S*,4*R*,5*S*,7*S*,8*R*,9*R*,16*S*,18*S,*19*S*-18-methyldeoxaphomin, and the metabolite was given the trivial name methyldeoxaphomin NPDG-E (**5**) (**Figure 1C**).

Metabolite **6** was obtained as a white powder. Its molecular formula, $C_{30}H_{39}NO_5$, was determined by the HRESIMS spectrum (*m/z* 516.2720 $[M+Na]^+$, calcd for $C_{30}H_{39}NNaO_5$, 516.2730). Considering the molecular formular and 1D and 2D NMR spectra of **6**, we deduced the structure was the reduced form of the ketone at C-20 in **2** and the oxidized form of C-19 in **5**, featuring hydroxy groups attached to both C-19 and C-20 (**Table S2 and Figure 1A**). The relative structure of **6** was also verified to be identical to the previously introduced compounds **1**-**5** through the ROESY correlations (JBCA and

Mosher's method) (**Figure 1B**). Based on the negative Cotton effects at 211 nm and the positive Cotton effects at 255 nm and 324 nm, the absolute structure of **6** was established as 13*E*,21*E*-3*S*,4*R*,5*S*,7*S*,8*R*,9*R*,16*R*,18*S*,19*S*,20*S*-18-methyl-19- deoxaphomin, and the metabolite was given the trivial name methyldeoxaphomin NPDG-F (**6**) (**Figure 1C**).

Plasmodium blood stage culture

Parasite culture in the Chakrabarti lab was maintained following a modified Trager and Jensen protocol (Trager & Jensen, 1976) as previously described (Lee et al., 2021) with RPMI 1640 with 25 mM HEPES pH 7.4, 26 mM $NaHCO_3$, 15 mg/L hypoxanthine, 0.5% Albumax II, 25 mg/L gentamycin, and 2% dextrose. Parasites were grown with human A+ erythrocytes obtained from Florida OneBlood. Cultures were kept in an incubator with 5% CO_2, 95% air at 37°C.

Antiplasmodial activity screening

SYBR Green I-based fluorescence assay was performed as previously described (Lee et al., 2021) following protocols by Smilkstein et. al. (Smilkstein et al., 2004). Asynchronous Dd2 parasites were added to serially diluted compounds and incubated for 72 h at 5% CO_2, 95% air at 37°C. Plates were then frozen at -80°C, thawed, and incubated for one hour with 1x SYBR Green I in a lysis buffer (20 mM Tris-HCL, 0.08% saponin, 5 mM EDTA, and 0.8% Triton X-100). Fluorescence was then read on a Synergy Neo2 multimode reader (BioTek Winsooki, VT). Compound dose response curves and EC_{50} determination was performed using CDD Vault normalized to 10 μM CQ and vehicle controls.

Isobologram assay

For isobologram assessment, inhibitors were tested using a 1:2 dilution with starting concentrations selected as 40 nM for DHA, 100 μM Latrunculin B, 100 μM SMIFH2, and 40 nM for jasplakinolide. These concentrations were selected to provide 2-3 points above

the inhouse measured EC_{50}s of these compounds, and 2-3 points below the measured EC_{50}s. The test inhibitor methyldeoxaphomin NPDG-F was then added in combination with the aforementioned inhibitors at concentrations ranging from 10 to 0.3125 μM. Dose-response curves were then generated for the available conditions where EC_{50}s could be determined. The fractional inhibitor concentration of both compounds was then determined as described (Fivelman et al., 2004) for the available points and graphed on a scale of 0-1 also using Graphpad Prism version 10.

Cytotoxicity screening

Cytotoxicity was assessed using an MTS (3-(4,5-Dimethylthiazol-2-yl)-5-(3-carboxymethoxyphenyl)-2-(4-sulfophenyl)-2H-tetrazolium) absorbance assay with human HepG2 hepatoma cells. Cells were maintained in MEM supplemented with 10%FBS, 1% Antibiotic/Antimycotic, 1 mM sodium pyruvate, and 0.15% sodium bicarbonate in a 5 % CO_2, 95% air environment at 37°C. For the assay cells were seeded at a density of 2,250 cells per well on 384-well microtiter plates 24 h prior to compound addition. Following a 48-h incubation with compounds, MTS solution was added to wells and absorbance was read after 2-4 h on a Synergy Neo2 multimode reader (BioTek Winsooki, VT). Compound dose response curves and EC_{50} determination was performed using CDD Vault normalized to 5% triton and vehicle controls.

Stage specific activity assay

Prior to compound addition, Dd2 culture was synchronized through combination of MACS column (Mata-Cantero et al., 2014) and sorbitol treatment (Lambros & Vanderberg, 1979). Compound was then added to synchronous culture as previously described (Collins et al., 2021) at 1% parasitemia, 2% hematocrit. In brief, culture was added to a microtiter plate, and compound or vehicle was added to different wells at either 6, 18, 30, or 42 HPI at a 5 x EC_{50} concentration. Every 12 h, culture was collected for flow-cytometric analysis with YOYO-1 and staining with Giemsa. Cultured was gated through comparison to uninfected RBCs, unstained infected RBCs, and stained vehicle controls using Flowjo version 10.

Rate of killing assay

Following the previously described protocol (Collins et al., 2021), compound or vehicle control was added to Dd2 culture in the majority ring stage and incubated for either 12, 24, or 48 h prior to compound removal. Samples were then taken at the time of compound removal, and every 24 h for 6 days for flow analysis with SYBR Green I and Mitotracker Deep red FM. Analysis was performed in Flowjo version 10 with gating using DHA, vehicle, no mitotracker, no staining, and uninfected RBC controls. Cells designated as SYBR positive and mitotracker positive were gated as “viable parasites” and graphed using Graphpad Prism version 10.

Parasite reduction ratio

Parasite reduction ratio adapted from protocol by Sanz et al (Sanz et al., 2012). In brief, early ring stage mono-infected RBCs at 10^6 were incubated with a 10 x EC_{50} concentration of NPDG-F, DHA, or atovaquone for either 24, 48, 72, 96, or 120 h. Following incubations, cells were washed and serially diluted. After 3 weeks, growth was determined via SYBR Green I staining. The PRR was calculated and graphed using Graphpad Prism version 10.

High content imaging liver stage screening

To examine the impact of methyldeoxaphomin NDPG F on the Plasmodium liver stage, HepG2 cells were infected with a luciferase-expressing strain of *P. berghei*. Following sporozoite addition, cells were seeded 2 h later onto a 384-well plate containing varying concentrations of the inhibitor. At 48 HPI, luciferase expression was measured as described(Van Voorhis et al., 2016) and parasite growth was quantified. After 72 HPI, High content imaging was performed to observe the relative expression of MSP1 and AMA1, the size and quantity of exoerythrocytic form parasites, and the HepG2 area occupied. Data was normalized to in-plate DMSO controls.

***Plasmodium in vitro* evolution of resistance**

The *in vitro* evolution was conducted using *Plasmodium falciparum* Dd2-Pol δ. This mutator strain with altered catalytic residues in DNA polymerase δ was developed via CRISPR-Cas9 to facilitate faster mutation rate and shorter selection period (Kumpornsin et al., 2023). Parasites were cultured in human O-positive blood cells with leukocyte depleted obtained from BioIVT (Westbury, NY). Blood was washed 3 times with RPMI 1640 (Gibco) upon arrival to rid of any remaining platelets and leukocytes and stored at 4 °C at 100% hematocrit. Parasites were cultured in T75 flasks with 40 ml of media at 2% hematocrit. A single parenteral line (control) and 3 drug-treated parasite lines (3 biological replicates) were maintained at any given time. Every 2 or 3 days, parasitemia was determined via Giemsa smear and reset to 2% (for a 3-day split, 1.6 $X10^8$ parasites) or 3% (for a 2-day split, 2.4 $X10^8$). Parasites were replenished with fresh blood and fresh RPMI 1640 media (Gibco) supplemented with 0.25% Albumax II (Gibco), 26 mM Sodium bicarbonate (Sigma), 0.1 mM hypoxanthine (Sigma), and 50 µg/L gentamicin (Gibco). The appropriate amount of the compound was added. Parasites were incubated at 37°C in sealed flasks filled with a mixture of gases containing 5% CO_2, 3% O_2, and 92% N_2. In this manner, parasites were cultured continuously with increasing drug concentration from 90 nM to 17 µM for 6.3 months. Resistance development was monitored every 3 months through SYBR green I- based EC_{50} dose response assay as described above.

Whole genome sequencing analysis

Parasite genomic DNA was extracted from the parent and the resistant lines using DNeasy Blood & Tissue Kit (Qiagen). DNA libraries were generated using Nextera XT DNA Library Preparation Kit (Illumina). The pooled libraries were sequenced on Illumina NovaSeq 6000 with S4 200 chemistry to generate paired end reads with 100bp in length. Reads were aligned to *Plasmodium falciparum* reference genome 3D7 (PlasmoDB v13.0) using an in-house pipeline (Cowell & Winzeler, 2019). SNVs and INDELs were identified using GATK HaplotypeCaller and filtered based on the GATK recommended depth and

quality thresholds. CNVs were assessed by running GATK 4.0 CollectReadCounts using all gene intervals and denoising according to a 3D7 panel of normals (constructed from non-drug-selected 3D7 parents). Genes with log2 denoised copy ratio of > 0.6 were considered to have amplification (copy gain), while those with copy ratio < -0.6 were considered to have deamplification (copy loss).

Trichocladium asperum gDNA was extracted using Quick-DNA™ Fungal/Bacterial Kits (Zymo Research), from which a DNA library was generated using Nextera XT DNA Library Preparation Kit (Illumina). This DNA library was sequenced with Illumina NovaSeq X Plus on a 10B flow cell to generate paired end reads ~100bp in length. Since a reference genome did not exist for *Trichocladium asperum* at the time of this study, to identify the contig containing the *Trichocladium asperum* actin gene, the WGS reads were aligned to ASPACDRAFT_1882512 (actin gene of *Aspergillus aculeatus* strain ATCC 16872, sequence accessed via FungiDB) using BWA-MEM with default parameters. Mapped reads were assembled using a custom Python script, and UTRs and introns were inferred based on close homology to the *Aspergillus aculeatus* actin gene. Upon removing UTRs and introns, the coding sequence for the *Trichocladium asperum* gene was obtained, encoding an actin with 376 amino acids.

Limiting dilution to generate clonal lines

Parasites were diluted to 0.5 parasite per well into 96-well plates. Parasites were fed with fresh media every 2 or 3 days with the addition of fresh blood. Two weeks later, blood smears were made to determine parasitemia. Thereafter, parasitemia was monitored every 2 or 3 days until viable parasites were identified. Four clones from flask No.1 and 2 clones each from flasks No. 2 and 3 were selected due to a higher number of mutations in PfActin1 in flask No. 1. The resistance phenotype of these clones was measured via EC_{50} assay as described below. DNA of the clones was extracted, and the library was prepped and sent for sequencing using the method described above. Sequencing analysis was done using the Winzeler lab in-house pipeline as described earlier.

Resistant parasite phenotyping and cross resistance measurement

The EC_{50} assay was performed at 1% parasitemia, 1% hematocrit with SYBR green I in 96-well plates. The highest final concentration of the methyldeoxaphomin NPDG-F was set at 100 μM. Briefly, the compound was subjected to a 3-fold serial dilution with ten dilution points. Three technical replicates were set up at each dilution point. The Dd2-Pol δ parent line and the resistant parasites at 2% hematocrit and 1% parasitemia were added to the drug media at 1:1 ratio. The plate was incubated at 37°C for 72 hours in a chamber filled with a hypoxic gas mixture (3% oxygen, 5% carbon dioxide, and 92% nitrogen). After incubation, the cells were lysis with lysis buffer (20 mM Tris-HCL, 0.08% saponin, 5 mM EDTA, and 0.8% Triton X-100) containing SYBR green I (10,000X, Thermo Fisher, cat # S7563) overnight. Fluorescence was measured at 485nm excitation and 530nm emission using a PHERAstar FSX plate reader (BMG Labtech, Germany). Dose response curves were graphed with Prism 9 Version 9.5.1.

3D structural analysis of the mutations and docking studies

The crystal structure of PfActin1 with accession number 6TU4 (resolution 2.60 Ang) (Vahokoski et al., 2022) was obtained from the Protein Data Bank (PDB). This structure of PfActin1 contains ligands Jasplakinolide and ADP with a coordinating magnesium ion. Only chain A was used for the docking experiment. The 3D conformer of cytochalasin D was downloaded from Pubchem (PubChem CID: 5458428). This docking grid encompassing subdomain 1 and 3 was chosen considering the locations of the three mutations identified via *in vitro* evolution and whole genome sequencing. Schrodinger maestro software (Schrödinger, LLC) was used to build NPDG-F with the proper stereochemistry using the x-ray structure determined for analog NPDG-A (vide supra). Ligand preparation for docking was then carried out in LigPrep (Schrödinger, LLC) with macrocyclic conformer search. Thirty-two low energy conformations were generated. The ligand conformers and protein were then docked using Glide SP (Schrödinger, LLC) . The exhaustiveness was set at 18 and num_modes was set at 5 per conformer. All poses were visually examined, and the docking poses for NPDG-F and cytochalasin D with at

least one hydrogen bond to Ile-137 backbone amine were selected with a docking score of -6.8 kcal/mol and -5.4 respectively.

In addition, a crystal structure of *Drosophila melanogaster* (Dm) actin (PDB No. 3EKS) (Nair et al., 2008) with cytochalasin D bound was utilized to showcase the hydrogen bonds. The human skeletal a-actin (PDB No. 6VAO) (Huehn et al., 2020) was predicted to be most similar to PfActin-1 using a BLAST sequence search with an identity of 82 % and similarity of 92% on protein level. All residues surrounding NPDG-F within 4-5 Ang of PfActin1 were identified and assessed visually. An Emboss Needle (EMBL-EBI) pairwise alignment was calculated, and binding site residues were highlighted in the sequence alignment. A comparison of the PfActin1 (6TU4) primary sequence was compared to human skeletal a-actin structure (PDB No. 6VAO) and *Tricocladium asperum* actin (in-house sequencing data). The corresponding residues in human and *T. asperum* actins lining the binding pockets were highlighted.

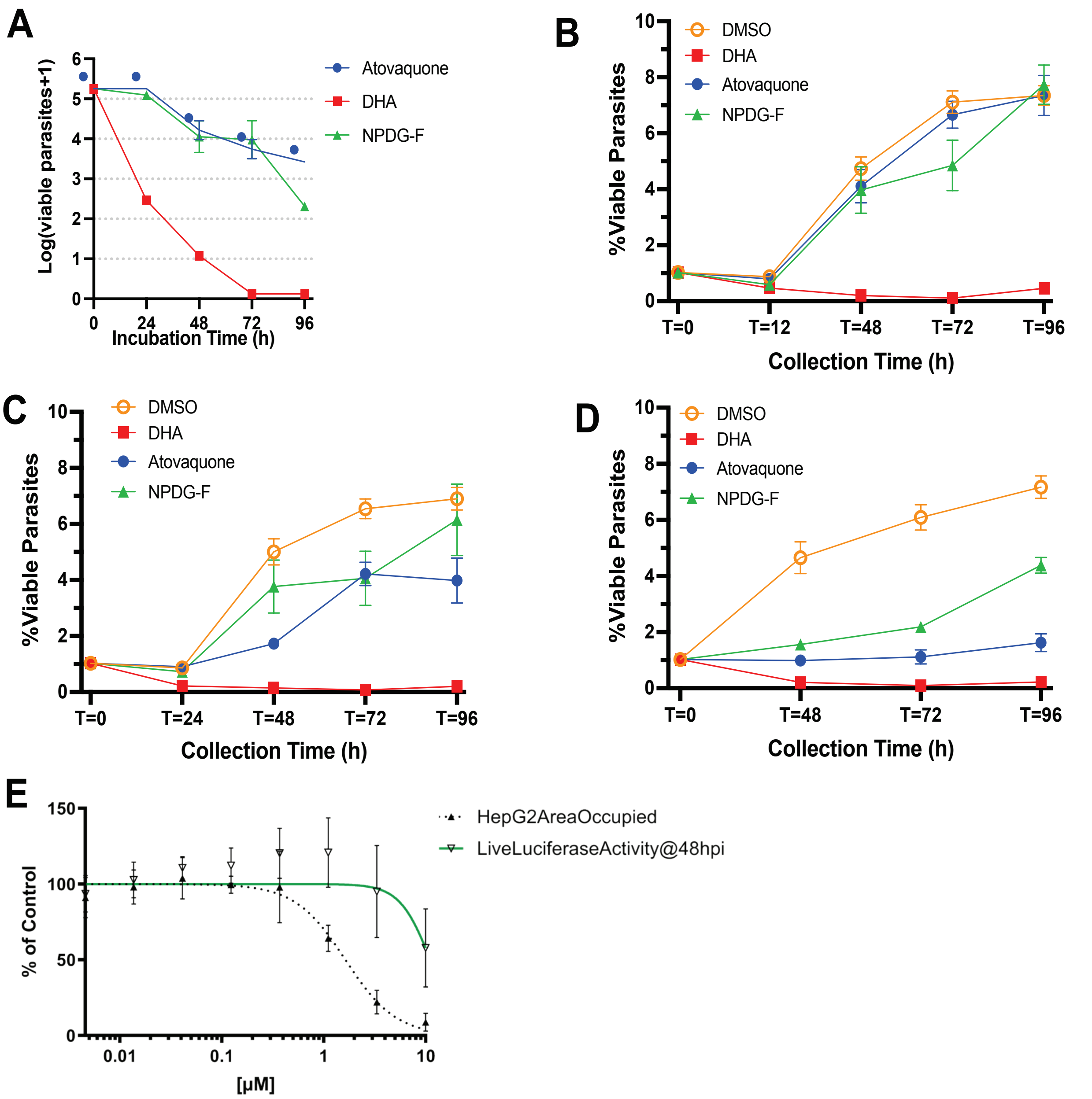

Figure S1. Killing rate profile of methyldeoxaphomin NPDG-F. (A) Parasite Reduction Ratio (PRR) of NPDG-F. Results represent the mean and SEM of two biological replicates. **(B)** Parasitemia following incubation of NPDG-F, DHA, or atovaquone at 10 x EC_{50} for 12 h, **(C)** 24 h, or **(D)** 48 HPI. Percent viable parasites were calculated based on SYBR Green I and Mitrotraker Deep Red FM positive parasites detected via flow cytometry with 100,000 events. Results represent the mean and SEM of three biological replicates. **(E)** HepG2 cells were infected with luciferase-expressing *P. berghei* sporozoites. Bioluminescent signal was taken at 48HPI.

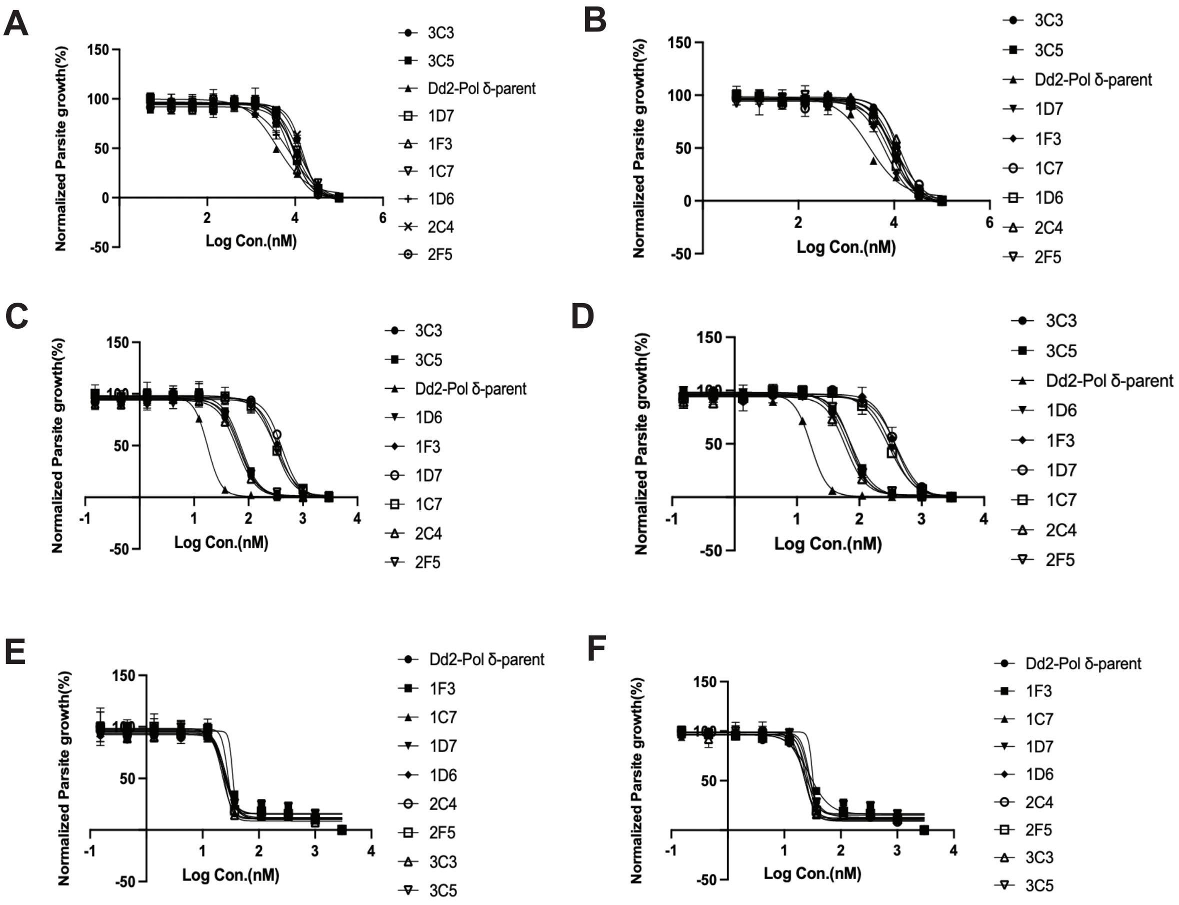

Figure S2. Phenotyping of the resistant clones and the cross-resistance tests to cytochalasin D and jasplakinolide. (A and B) Two biological replicates of the EC_{50} dose response assay of the clones with methyldeoxaphomin NPDG-F. Each biological replicate contains two technical replicates. EC_{50} values and fold change were shown in Table 2. **(C and D)** Two biological replicates of the cross-resistance of the clones to cytochalasin D. Each biological replicate contains two technical replicates. **(E and F)** Two biological replicates of the cross-resistance of the clones to jasplakinolide. Each biological replicate contains two technical replicates.

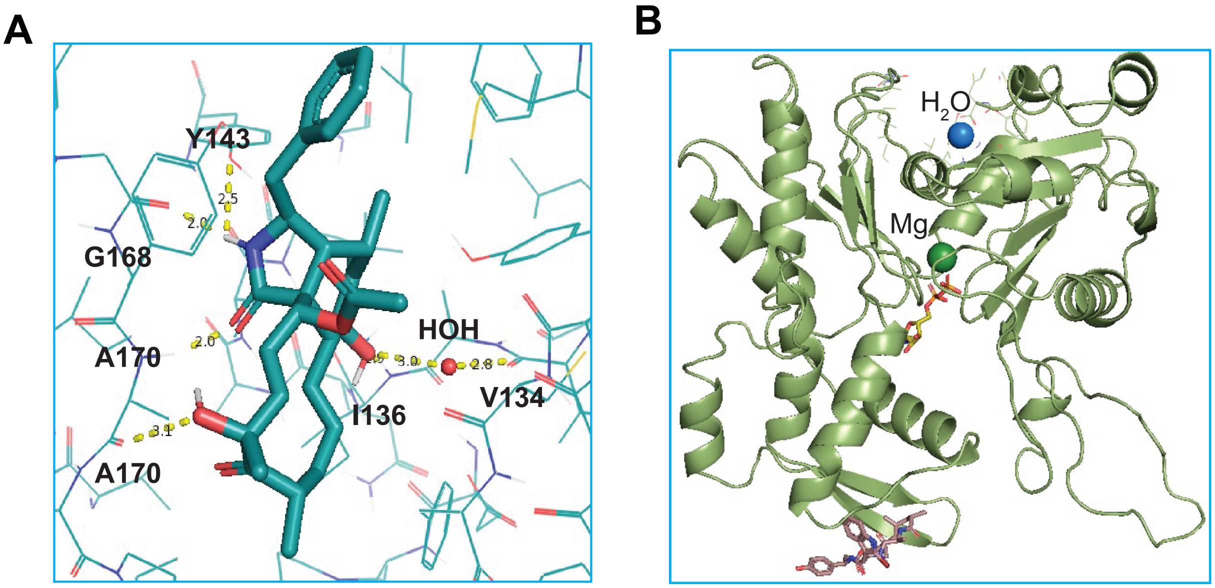

C

```
Tricocladium_actin     QSKRGILTLRYPIEHGVVTNWDDMEKIWHHTFYNELRVAPEEHPVLLTEAPINPKSNREK  118
PfActin1_6TU4          QTKRGILTLKYPIEHGIVTNWDDMEKIWHHTFYNELRAAPEEHPVLLTEAPLNPKGNRER  119
Human_actin_6VAO       QSKRGILTLKYPIEHGIITNWDDMEKIWHHTFYNELRVAPEEHPTLLTEAPLNPKANREK  120
                       *.*******:******::*******************.******.******:***.***:

Tricocladium_actin     MTQIVFETFNAPAFYVSIQAVLSLYASGRTTGIVLDSGDGVTHVVPIYEGFALPHAISRV  178
PfActin1_6TU4          MTQIMFESFNVPAMYVAIQAVLSLYSSGRTTGIVLDSGDGVSHTVPIYEGYALPHAIMRL  179
Human_actin_6VAO       MTQIMFETFNVPAMYVAIQAVLSLYASGRTTGIVLDSGDGVTHNVPIYEGYALPHAIMRL  180
                       ****:**.**.**.**.********.***************.* ******:******.*:
                                     A136                                A171

Tricocladium_actin     DMAGRDLTDYLMKILAERGYTFSTTAEREIVRDIKEKLCYVALDFEQEIQTASQSSSLEK  238
PfActin1_6TU4          DLAGRDLTEYLMKILHERGYGFSTSAEKEIVRDIKEKLCYIALNFDEEMKTSEQSSDIEK  239
Human_actin_6VAO       DLAGRDLTDYLMKILTERGYSFVTTAEREIVRDIKEKLCYVALDFENEMATAASSSSLEK  240
                       *:******:****** **** *.*.**:************:**.*:.*: *. .**..**

Tricocladium_actin     SYELPDGQVITIGNERFRAPEALFQPSVLGLESGGIHVTTFNSIMKCDVDVRKDLYGNIV  298
PfActin1_6TU4          SYELPDGNIITVGNERFRCPEALFQPSFLGKEAAGIHTTTFNSIKKCDVDIRKDLYGNIV  299
Human_actin_6VAO       SYELPDGQVITIGNERFRCPETLFQPSFIGMESAGIHETTYNSIMKCDIDIRKDLYANNV  300
                       *******.:**:******.**:*****.:* *..*** **:***.***:*:*****.*.*

Tricocladium_actin     MSGGTTMYPGISDRMQKEITALAPSSMKVKIIAPPERKYSVWIGGSILASLSTFQQMWIS  358
PfActin1_6TU4          LSGGTTMYEGIGERLTRDITTLAPSTMKIKVVAPPERKYSVWIGGSILSSLSTFQQMWIT  359
Human_actin_6VAO       MSGGTTMYPGIADRMQKEITALAPSTMKIKIIAPPERKYSVWIGGSILASLSTFQQMWIT  360
                       :*******.**.:*:.::**.****.**:*::****************.***********.
```

D

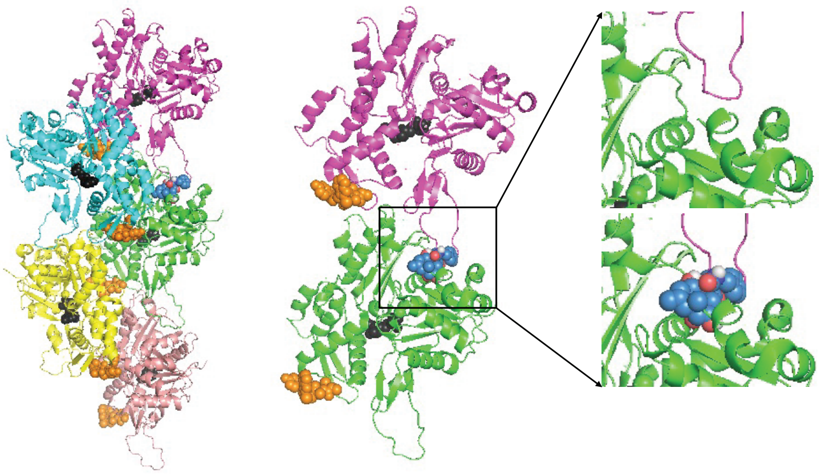

Figure S3. High similarity of the binding pockets of NPDG-F with human and *Plasmodium* actins. (A) The six hydrogen bonds identified in the co-crystallization of cytochalasin D with *Drosophila melanogaster* actin (3EKS), as previously described. **(B)** The water molecule found in the hydrophobic cleft between subdomains 1 and 3 of PfActin1 (6TU4). This water molecule was removed before docking. **(C)** The alignment of human skeletal alpha actin (6VAO), PfActin1 (6TU4), and *Tricocladium asperum* actin (in-house sequencing data) based on their primary structure. Residues lining the NPDG-F binding pocket in PfActin1 were boxed. **(D)** D-loop extends to the hydrophobic cleft between subdomains 1 and 3 but did not penetrate the cleft. The actin monomers are shown as cartoon with different colors. The ligands are shown as spheres (blue: NPDG-F; black: ADP; orange: jasplakinolide).

Table S1. ^{1}H (500 MHz) and ^{13}C NMR (100 MHz) data of compounds 1-3 (NPDG-A to C) in $CDCl_3$

No.	1		2		3	
	δ_H	δ_C	δ_H	δ_C	δ_H	δ_C
1	-	171.50	-	171.30	-	171.50
2	5.54 s	-	5.97 s	-	5.65 s	
3	3.41 ddd (10.0, 6.0, 3.5)	54.20	3.42 ddd (10.0, 6.0, 3.5)	54.51	3.35 t (7.5)	53.03
4	3.23 m	45.15	3.23 dd (6.0, 4.5)	44.73	2.43 d (6.0)	46.99
5	2.79 m	32.39	2.74 m	32.45	2.95 t (6.0)	31.76
6	-	148.55	-	148.63	-	146.80
7	4.03 dd (10.0, 2.0)	69.43	4.02 dd (10.0, 2.0)	69.24	4.03 d (10.0)	71.22
8	2.40 t (10.0)	49.97	2.34 t (10.0)	49.77	2.84 t (10.0)	47.99
9	-	62.30	-	62.22	-	61.30
10	3.01 dd (14.0, 3.5)<br>2.48 dd (14.0, 10.0)	44.67	3.04 dd (14.0, 3.5)<br>2.46 dd (14.0, 10.0)	44.73	2.58 m, 2.54 m	44.22
11	1.26 d (7.0)	15.75	1.29 d (6.5)	16.17	1.02 d (6.5)	13.08
12	5.47 br t (2.0)<br>5.19 br t (2.0)	112.84	5.48 br t (2.0)<br>5.19 br t (2.0)	112.57	5.33 br s<br>5.16 br s	115.86
13	5.96 dd (15.0, 10.0)	129.04	5.91, m	129.15	6.32 dd (15.5, 10.0)	128.50
14	5.83 ddd (15.0, 8.5, 6.0)	137.33	5.87 m	137.65	5.99 ddd (15.5, 8.0, 6.0)	137.19
15	2.28 m, 1.91 m	41.03	2.36 m<br>1.88 ddd (15.0, 10.0, 6.0)	41.60	2.31 dd (14.0, 8.0)<br>1.73 m	40.30
16	2.02 m	31.87	2.12 m	32.30	1.97 m	32.82
17	1.33 m (2H)	45.82	1.76 t (13.0)<br>1.16 ddd (13.0, 10.5, 3.0)	42.80	1.69 m<br>1.11 ddd (14.0, 11.5, 3.0)	43.65
18	1.94 m	30.61	2.03 m	35.80	2.03 m	34.41
19	3.24 m, 1.93 m	46.82	5.01 br s	74.51	4.55 br s	75.39
20	-	203.16	-	203.85	-	207.62
21	6.73 d (16.5)	137.31	6.85 d (16.5)	133.81	6.53 d (12.5)	141.90
22	7.81 d (16.5)	135.27	7.88 d (16.5)	135.96	6.77 d (12.5)	128.71
23	-	195.74	-	194.59	-	196.07
24	1.00 d (7.0)	23.75	1.03 d (6.5)	24.54	0.97 d (7.0)	24.77
25	0.90 d (6.5)	21.37	0.63 d (7.0)	14.15	0.69 d (7.0)	14.63
1'	-	137.12	-	137.07	-	136.64
2', 6'	7.14 m (2H)	128.94	7.14 m (2H)	128.96	7.08 m (2H)	129.14
3', 5'	7.32 m (2H)	129.04	7.31 m (2H)	129.02	7.30 m (2H)	128.95
4'	7.26 m	127.20	7.25 m	127.20	7.25 m	127.18

Table S2. ^{1}H (500 MHz) and ^{13}C NMR (100 MHz) data of compounds 4-6 (NPDG-D to F) in $CDCl_3$

No.	4		5		6	
	δ_H	δ_C	δ_H	δ_C	δ_H	δ_C
1	-	171.43	-	174.29	-	174.03
2	5.39 s	-	5.98 s	-	5.63 s	
3	3.36 m	55.51	3.35 m	53.25	3.35 t (6.5)	53.12
4	3.38 m	48.15	2.84 dd (6.0, 2.0)	46.86	2.84 d (6.0)	47.09
5	2.51 m	33.44	2.90 t (6.0)	31.77	2.93 t (6.0)	31.81
6	-	143.20	-	147.87	-	147.77
7	5.60 br s	128.13	3.98 d (10.0)	70.58	3.99 d (10.0)	70.56
8	2.63 d (10.0)	44.97	2.70 t (10.0)	50.17	2.73 t (10.0)	50.35
9	-	63.64	-	61.72	-	61.69
10	3.10 dd (14.0, 3.5)<br>2.47 dd (14.0, 10.0)	44.93	2.64 dd (13.5, 5.5)<br>2.56 dd (13.5, 8.0)	43.99	2.66 dd (13.5, 5.5)<br>2.55 dd (13.5, 8.5)	44.07
11	1.44 d (7.5)	13.94	0.98 d (7.0)	13.16	1.01 d (6.5)	13.11
12	4.23 s (2H)	63.64	5.29 br s<br>5.10 br s	114.75	5.30 br s<br>5.12 br s	114.84
13	5.98 dd (15.0, 10.0)	131.97	6.11 dd (15.0, 10.0)	126.78	6.13 dd (16.0, 9.5)	126.98
14	5.74 ddd (15.0, 10.0, 6.0)	133.34	5.65 ddd (15.0, 10.0, 5.0)	136.10	5.64 ddd (16.0, 9.5, 5.0)	135.73
15	2.35 m, 1.81 m	42.09	2.12 ddd (13.5, 10.0, 3.5)<br>1.96 m	40.24	2.31 m, 2.03 m	39.43
16	2.10 m	32.91	1.71 m	30.40	1.83 m	29.46
17	1.79 m, 1.20 m	43.00	1.19 m<br>1.07 ddd (13.5, 8.5, 5.5)	43.29	1.34 ddd (13.5, 8.5, 4.0)<br>0.94 m	37.50
18	2.17 m	36.04	1.74 m	43.70	1.81 m	33.49
19	5.04 d (5.5)	74.33	1.77 m	26.66	3.73 d (6.5)	79.04
20	-	203.99	4.55 m	70.39	4.49 d (6.5)	74.38
21	6.86 d (16.5)	133.31	6.81 dd (15.5, 5.5)	151.06	6.98 dd (15.5, 6.5)	146.09
22	8.01 d (16.5)	136.43	6.85 d (15.5)	124.74	6.90 d (15.5)	125.73
23	-	195.33	-	197.55	-	197.45
24	1.04 d (6.5)	24.83	0.88 d (7.0)	20.63	0.91 d (6.5)	19.51
25	0.64 d (6.5)	14.13	0.96 d (7.0)	21.07	1.00 d (7.0)	16.28
1'	-	137.41	-	137.00	-	137.03
2', 6'	7.13 m (2H)	128.82	7.11 m (2H)	129.28	7.12 m (2H)	129.24
3', 5'	7.31 m (2H)	129.05	7.30 m (2H)	128.83	7.30 m (2H)	128.87
4'	7.25 m	127.14	7.24 m	126.97	7.25 m	127.00

Movie S1 (separate file). Live imaging of merosomes taken at 72 HPI. This is a video representation of Figure 4H(ii), showing the strong inhibition of merozoite formation. Scale bars = 5μm

Movie S2 (separate file). Live imaging of merosomes taken at 72 HPI. This is a video representation of Figure 4H(iii), showing the weak inhibition of merozoite formation. Scale bars = 5μm

Dataset S1 (separate file). The antimalarial activities of the six compounds and their cytotoxicity

Dataset S2 (separate file). The copy number variant (CNVs) analysis of the parent line and the resistant clones

Dataset S3 (separate file). The single nucleotide variant analysis (SNVs) of the parent line and the resistant clones

SI References

Chen, Z. M., Chen, H. P., Li, Y., Feng, T., & Liu, J. K. (2015). Cytochalasins from cultures of endophytic fungus Phoma multirostrata EA-12. *J Antibiot (Tokyo)*, *68*(1), 23-26. https://doi.org/10.1038/ja.2014.87

Collins, J. E., Lee, J. W., Bohmer, M. J., Welden, J. D., Arshadi, A. K., Du, L., Cichewicz, R. H., & Chakrabarti, D. (2021). Cyclic Tetrapeptide HDAC Inhibitors with Improved Plasmodium falciparum Selectivity and Killing Profile. *ACS Infect Dis*, *7*(10), 2889-2903. https://doi.org/10.1021/acsinfecdis.1c00341

Cowell, A. N., & Winzeler, E. A. (2019). Advances in omics-based methods to identify novel targets for malaria and other parasitic protozoan infections. *Genome Med*, *11*(1), 63. https://doi.org/10.1186/s13073-019-0673-3

Fivelman, Q. L., Adagu, I. S., & Warhurst, D. C. (2004). Modified fixed-ratio isobologram method for studying in vitro interactions between atovaquone and proguanil or dihydroartemisinin against drug-resistant strains of Plasmodium falciparum. *Antimicrob Agents Chemother*, *48*(11), 4097-4102. https://doi.org/10.1128/AAC.48.11.4097-4102.2004

Hu, X. Y., Li, X. M., Yang, S. Q., Wang, B. G., & Meng, L. H. (2022). New Cytochalasin Derivatives from Deep-Sea Cold Seep-Derived Endozoic Fungus Curvularia verruculosa CS-129. *Chem Biodivers*, *19*(8), e202200550. https://doi.org/10.1002/cbdv.202200550

Huehn, A. R., Bibeau, J. P., Schramm, A. C., Cao, W., De La Cruz, E. M., & Sindelar, C. V. (2020). Structures of cofilin-induced structural changes reveal local and asymmetric perturbations of actin filaments. *Proc Natl Acad Sci U S A*, *117*(3), 1478-1484. https://doi.org/10.1073/pnas.1915987117

Kim, E. L., Li, J. L., Dang, H. T., Hong, J., Lee, C. O., Kim, D. K., Yoon, W. D., Kim, E., Liu, Y., & Jung, J. H. (2012). Cytotoxic cytochalasins from the endozoic fungus Phoma sp. of the giant jellyfish Nemopilema nomurai. *Bioorg Med Chem Lett*, *22*(9), 3126-3129. https://doi.org/10.1016/j.bmcl.2012.03.058

Kumpornsin, K., Kochakarn, T., Yeo, T., Okombo, J., Luth, M. R., Hoshizaki, J., Rawat, M., Pearson, R. D., Schindler, K. A., Mok, S., Park, H., Uhlemann, A. C., Jana, G. P., Maity, B. C., Laleu, B., Chenu, E., Duffy, J., Moliner Cubel, S., Franco, V., . . . Lee, M. C. S. (2023). Generation of a mutator parasite to drive resistome discovery in Plasmodium falciparum. *Nat Commun*, *14*(1), 3059. https://doi.org/10.1038/s41467-023-38774-1

Lambros, C., & Vanderberg, J. P. (1979). Synchronization of Plasmodium falciparum erythrocytic stages in culture. *J Parasitol*, *65*(3), 418-420. https://www.ncbi.nlm.nih.gov/pubmed/383936

Lee, J. W., Collins, J. E., Wendt, K. L., Chakrabarti, D., & Cichewicz, R. H. (2021). Leveraging Peptaibol Biosynthetic Promiscuity for Next-Generation Antiplasmodial Therapeutics. *J Nat Prod*, *84*(2), 503-517. https://doi.org/10.1021/acs.jnatprod.0c01370

Mata-Cantero, L., Lafuente, M. J., Sanz, L., & Rodriguez, M. S. (2014). Magnetic isolation of Plasmodium falciparum schizonts iRBCs to generate a high parasitaemia and synchronized in vitro culture. *Malar J*, *13*, 112. https://doi.org/10.1186/1475-2875-13-112

Meng, X., Fang, Y., Ding, M., Zhang, Y., Jia, K., Li, Z., Collemare, J., & Liu, W. (2022). Developing fungal heterologous expression platforms to explore and improve the production of natural products from fungal biodiversity. *Biotechnol Adv*, *54*, 107866. https://doi.org/10.1016/j.biotechadv.2021.107866

Nair, U. B., Joel, P. B., Wan, Q., Lowey, S., Rould, M. A., & Trybus, K. M. (2008). Crystal structures of monomeric actin bound to cytochalasin D. *J Mol Biol*, *384*(4), 848-864. https://doi.org/10.1016/j.jmb.2008.09.082

Robert, J. L., & Tamm, C. (1975). Biosynthesis of cytochalasans. Part 5. The incorporation of deoxaphomin into cytochalasin B (phomin). *Helv Chim Acta*, *58*(8), 2501-2504. https://doi.org/10.1002/hlca.19750580830

Sanz, L. M., Crespo, B., De-Cozar, C., Ding, X. C., Llergo, J. L., Burrows, J. N., Garcia-Bustos, J. F., & Gamo, F. J. (2012). P. falciparum in vitro killing rates allow to discriminate between different antimalarial mode-of-action. *PLoS One*, *7*(2), e30949. https://doi.org/10.1371/journal.pone.0030949

Smilkstein, M., Sriwilaijaroen, N., Kelly, J. X., Wilairat, P., & Riscoe, M. (2004). Simple and inexpensive fluorescence-based technique for high-throughput antimalarial drug screening. *Antimicrob Agents Chemother*, *48*(5), 1803-1806. https://www.ncbi.nlm.nih.gov/pubmed/15105138

Trager, W., & Jensen, J. B. (1976). Human malaria parasites in continuous culture. *Science*, *193*(4254), 673-675. https://www.ncbi.nlm.nih.gov/pubmed/781840

Vahokoski, J., Calder, L. J., Lopez, A. J., Molloy, J. E., Kursula, I., & Rosenthal, P. B. (2022). High-resolution structures of malaria parasite actomyosin and actin filaments. *PLoS Pathog*, *18*(4), e1010408. https://doi.org/10.1371/journal.ppat.1010408

Van Voorhis, W. C., Adams, J. H., Adelfio, R., Ahyong, V., Akabas, M. H., Alano, P., Alday, A., Aleman Resto, Y., Alsibaee, A., Alzualde, A., Andrews, K. T., Avery, S. V., Avery, V. M., Ayong, L., Baker, M., Baker, S., Ben Mamoun, C., Bhatia, S., Bickle, Q., . . . Willis, P. A. (2016). Open Source Drug Discovery with the Malaria Box Compound Collection for Neglected Diseases and Beyond. *PLoS Pathog*, *12*(7), e1005763. https://doi.org/10.1371/journal.ppat.1005763
